# Comparative Transcriptome Analysis Reveals Critical Function of Sucrose Metabolism Related-Enzymes in Starch Accumulation in the Storage Root of Sweet Potato

**DOI:** 10.3389/fpls.2017.00914

**Published:** 2017-06-22

**Authors:** Kai Zhang, Zhengdan Wu, Daobin Tang, Kai Luo, Huixiang Lu, Yingying Liu, Jie Dong, Xin Wang, Changwen Lv, Jichun Wang, Kun Lu

**Affiliations:** ^1^College of Agronomy and Biotechnology, Southwest UniversityChongqing, China; ^2^Engineering Research Center of South Upland Agriculture, Ministry of Education, Southwest UniversityBeibei, China; ^3^Sweet Potato Engineering and Technology Research CenterChongqing, China

**Keywords:** expression profile, RNA-seq, starch, storage root, sucrose, sweet potato

## Abstract

The starch properties of the storage root (SR) affect the quality of sweet potato (*Ipomoea batatas* (L.) Lam.). Although numerous studies have analyzed the accumulation and properties of starch in sweet potato SRs, the transcriptomic variation associated with starch properties in SR has not been quantified. In this study, we measured the starch and sugar contents and analyzed the transcriptome profiles of SRs harvested from sweet potatoes with high, medium, and extremely low starch contents, at five developmental stages [65, 80, 95, 110, and 125 days after transplanting (DAP)]. We found that differences in both water content and starch accumulation in the dry matter affect the starch content of SRs in different sweet potato genotypes. Based on transcriptome sequencing data, we assembled 112336 unigenes, and identified several differentially expressed genes (DEGs) involved in starch and sucrose metabolism, and revealed the transcriptional regulatory network controlling starch and sucrose metabolism in sweet potato SRs. Correlation analysis between expression patterns and starch and sugar contents suggested that the sugar–starch conversion steps catalyzed by sucrose synthase (SuSy) and UDP-glucose pyrophosphorylase (UGPase) may be essential for starch accumulation in the dry matter of SRs, and IbβFRUCT2, a vacuolar acid invertase, might also be a key regulator of starch content in the SRs. Our results provide valuable resources for future investigations aimed at deciphering the molecular mechanisms determining the starch properties of sweet potato SRs.

## Introduction

Sweet potato (*Ipomoea batatas* (L.) Lam.) is an important food crop that is widely grown throughout the world due to its stable yield, rich nutrient content, low input requirement, multiple uses, high yield potential, and adaptability under a range of environmental conditions (Ahn et al., 2010; Cervantes-Flores et al., 2011; Wang et al., 2011). Sweet potato is mainly grown for its edible starchy storage root (SR), and the formation and development of SRs is the most economically important physiological process in sweet potato production. This process includes the adventitious roots arising from vegetative cuttings, fibrous roots (FRs) development and some of FRs subsequently developing into SRs, which accompanied with SRs swell up and weight increases through accumulating photosynthates and massive filling with starch (Ravi et al., 2009; Firon et al., 2013).

Starch is the major component of the SR, accounting for 50–80% of its dry matter (Rukundo et al., 2013; Zhou et al., 2015). This high level of starch renders sweet potato a good source of carbohydrates, and an excellent raw material for starch-based industries and biofuel production. Indeed, sweet potato may even have a greater potential as an ethanol source than corn in some regions (Ziska et al., 2009; Nedunchezhiyan et al., 2012; Koçar and Civaş, 2013). The demand for ethanol is expected to more than double in the next decade (Demirbas, 2009). As producing biofuel from biomass offers a renewable approach for reducing the consumption of crude oil, greenhouse gas emissions, and other environmental pollutants, and thus for offsetting climate change, global warming, and air pollution (Demirbas, 2009; Jacobson, 2009), methods to improve the quality of sweet potato as a feedstock for ethanol production should be investigated.

The quality of sweet potato as a feedstock for ethanol production and starch-based industries is determined by its starch properties. The dry matter content and starch content of the SR influence post-harvest processing and ethanol yield; thus, developing varieties of sweet potato with high levels of dry matter and starch in the SRs is an important target of sweet potato breeding programs (Tanaka et al., 2009; Nedunchezhiyan et al., 2012; Rukundo et al., 2013; Zhang et al., 2016). The composition of starch in the SR, particularly the ratio of amylose to amylopectin, also influences the physicochemical properties of starch (Hamada et al., 2006; Zhou et al., 2015) and ethanol yield (Nedunchezhiyan et al., 2012). However, the molecular mechanisms underlying the establishment and regulation of these traits in sweet potato was hitherto unclear. Furthermore, the dry matter content (ranging from 18 to 42%) and starch content of the SRs varies greatly among sweet potato genotypes (Li and Zhang, 2003; Ravi et al., 2009; Tumwegamire et al., 2011), but the genetic mechanism contributing to this variation was unclear.

Starch is the most important carbohydrate storage reserve in plants (Lai et al., 2016) and the major carbohydrate of tuber and root crops (Hoover, 2001). It is synthesized in higher plants through a complex pathway regulated by multiple starch-synthesizing enzymes (Lai et al., 2016). Genes that function in starch biosynthesis and metabolism have been studied in many higher plants (Zeeman et al., 2010). In sweet potato, the key enzymes involved in starch biosynthesis, including ADP-glucose pyrophosphorylase (AGPase, EC 2.7.7.27), granule-bound starch synthases (GBSS, EC 2.4.1.21), isoamylase (ISA, EC 3.2.1.68), starch-branching enzyme (SBE, EC 2.4.1.18), starch phosphorylase (SP, EC 2.4.1.1), and soluble starch synthase (SSS, EC 2.4.1.21), and their associated genes have been isolated and studied (Lin et al., 1991; Bae and Liu, 1997; Harn et al., 2000; Lee et al., 2000; Kim et al., 2005; Hamada et al., 2006; Ahn et al., 2010; Takahata et al., 2010; Qin et al., 2013; Lai et al., 2016). Several genes were confirmed to influence starch content or composition in RNA interference studies (Shimada et al., 2006; Kitahara et al., 2007; Otani et al., 2007). The starch content is reduced in the SRs of transgenic sweet potato plants in which the expression of *SBEII* (Shimada et al., 2006) or *GBSSI* (Otani et al., 2007) is suppressed by RNA interference. However, these studies did not provide insight into the biochemical and molecular mechanisms underlying starch properties.

In SRs, starch is synthesized from the cleaved products of imported photoassimilate sucrose from photosynthetic organs (Li and Zhang, 2003). Sucrose and other sugars not only serve as substrates for starch production, but also function as a signal that regulates SR development and various metabolic processes (Ravi et al., 2009). Sucrose metabolism thus influences starch synthesis and SR development, and genes involved in the starch–sugar interconversion also affect starch and sugar related traits in plant storage organs (Schreiber et al., 2014). However, little is known about sucrose metabolism and starch–sugar interconversion in the SR of sweet potato. Knowledge of the gene networks controlling starch biosynthesis and accumulation, sucrose metabolism, and starch–sugar interconversion would provide important insights into the mechanisms underlying starch properties in sweet potato SRs.

In this study, we selected three sweet potato varieties with different starch properties, monitored the accumulation and composition of starch and sugar during SR development, and used Illumina paired-end sequencing technology to perform an RNA-Seq analysis of the SRs at different developmental stages. We identified DEGs involved in starch biosynthesis and sucrose metabolism, and confirmed their expression profiles by quantitative real-time PCR (qRT-PCR). These results improve our understanding of the biochemical and molecular mechanisms underlying starch accumulation and starch–sugar interconversion during SR development in sweet potato.

## Materials and methods

### Plant material preparation

Three sweet potato varieties with different SR starch properties (i.e., Yushu 33 (YS33), Xushu 22 (XS22), and Shangqiu 52-7 (SQ52-7)) were selected for transcriptional profiling. Plants of the three varieties were cultivated at the experimental station of the Sweet Potato and Potato Research Institute, Southwest University, Chongqing, China, in 2013 and 2014. For each variety, fifty plants were produced from transplants and grown in unfertilized washed sand in the greenhouse, at temperatures of between 22 and 28°C. After transplanting into the field, roots from five individual plants were sampled at 50, 65, 80, 95, 110, 125, and 140 days after transplanting (DAP) and were divided in two samples; one sample was used to quantify starch properties (including starch and sugar content and composition) and the other one was immediately frozen in liquid nitrogen and used for RNA extraction. Samples were collected and assessed in triplicate each year. Fifteen samples, including the SRs sampled at 65, 80, 95, 110, and 125 DAP from YS33, XS22, and SQ52-7 in 2013 were used for transcriptome analysis.

### RNA extraction

Total RNAs were extracted from the root tissues and residual DNA was digested using the RNAprep Pure Plant Kit with DNase I (DP432, Tiangen Biotech, Beijing, China) according to the manufacturer's instructions. The total RNA samples were examined by agarose gel electrophoresis, and the concentration and quality of RNA were determined with a NanoDrop ND-2000 Spectrophotometer (Thermo Scientific, Waltham, MA, USA). RNA quality was verified using a 2100 Bioanalyzer RNA Nanochip (Agilent Technologies, Santa Clara, CA) and all the samples had an RNA Integrity Number (RIN) of > 8.5. The RNA samples were used for transcriptional profiling and qRT-PCR analysis. A total of 20 μg of RNA from each sample was used for cDNA library preparation.

### Quantification of SR starch and sugar properties

The dry matter, starch, amylose, and sugar contents of the SRs were determined for each sample. Dry matter was measured as previously described (Cervantes-Flores et al., 2011). The total starch was extracted and purified as previously described (Huang et al., 2010). Briefly, the dry matter was desugared and defatted by dissolving SR tissue in petroleum benzine and precipitating the solution with ethanol. The total starch content of the SRs was represented as a percentage of total fresh weight (FW). The amylose content and the ratio of amylose to amylopectin in the SR starch was estimated as we previously reported (Zhang et al., 2016). The amylose content was represented as a percentage of the dry weight of total starch. Sugar components were qualified at Zoonbio Biotechnology Co., Ltd, Nanjing, China, using high performance liquid chromatography (HPLC).

All of the statistical analyses were performed using Prism 7.0a software (GraphPad Software, La Jolla, CA, USA). For each trait, a two-way analysis of variance (ANOVA) was used to determine the effect of the genotype and developmental stage as well as their interaction. A Tukey's multiple comparison test was then employed to determine the statistical significance between varieties at each of the five developmental stages. Statistical comparisons were considered significant at *P* < 0.05.

### Sequencing and *de novo* assembly

Sequencing libraries of the 15 SR samples were constructed and Illumina paired-end (PE) sequencing using the Hiseq2000 platform was performed at Beijing Yuanquanyike Biotech, Beijing, China, according to the manufacturer's instructions (Illumina, San Diego, CA). To obtain clean reads, all of the raw reads were filtered with the following process. Firstly, reads that failed the built-in Failed Chastity Filter in the Illumina software according to the relation “failed-chastity ≤ 1,” using a chastity threshold of 0.6, on the first 25 cycles were excluded. Secondly, reads with adaptor contamination were discarded. Thirdly, low-quality reads were masked with ambiguous sequences “N.” Finally, reads with more than 10% *Q* < 20 were removed. All the filtered reads from the 15 libraries were *de novo* assembled using Trinity (RRID: SCR_013048, ver. trinityrnaseq_r2013_08_14) with paired-end method and default parameters as previous study on optimal assembly strategy (He et al., 2015).

### Unigene expression analysis and DEG identification

Genes differentially expressed between different genotypes at the same developmental stage, and between different developmental stages from the same genotype were screened. Firstly, we quantified the expression level of unigenes in the 15 samples. All the filtered reads were mapped to the unigenes in the newly assembled transcriptome using bowtie2-2.1.0 (RRID: SCR_005476). The unigene expression levels were quantified and normalized using the CLC Genomics Workbench version 3.7.1 (CLC Bio, Arhus, Denmark) in terms of reads per kilobase of exon model per million mapped reads (RPKM). Then, this commercially available software was used to test for statistically significant differences in unigene expression between two samples. DEGs were screened based on the following combined criteria: *q*-value [false discovery rate (FDR)] < 0.01 and absolute fold change of RPKM > 2.

### Cluster and correlation analyses of transcriptomes

The expression levels of all of the unigenes in the 15 samples were subjected to correlation analysis. The Pearson's correlation coefficient between samples was calculated and plotted using the “corrplot” package in R. For the 15 transcriptomes, heatmap plotting and hierarchical cluster analysis were performed using the heatmap.2 function of the “gplots” package in R, based on the normalized log2-transformed RPKM. For DEGs, the R package pheatmap was employed for heatmap generation.

### Functional annotation of unigenes by sequence comparison with public databases

BLASTx (RRID: SCR_001653) alignment (similarity>30%, *E* <1.0E-5) between unigenes and sequences derived from public databases, including the UniProtKB/Swiss-Prot Database (http://www.expasy.ch/sprot), TrEMBL Database (http://www.ebi.ac.uk/uniprot), the Conserved Domain Database (CDD, RRID: SCR_002077) (http://www.ncbi.nlm.nih.gov/cdd), the Pfam Database (RRID: SCR_004726) (http://pfam.xfam.org/), NCBI Non-redundant Protein (Nr) Database (http://www.ncbi.nlm.nih.gov), and the Eukaryotic Orthologous Groups (KOGs, RRID: SCR_006801) Database (http://www.ncbi.nlm.nih.gov/COG), was performed, and the best alignments were used to determine the sequence direction of unigenes.

### GO term and KEGG pathway enrichment

Gene ontology (GO) annotation analysis of the unigenes was performed using the high-score BLAST matches in the Swiss-Prot and TrEMBL Proteins Database (*E* <1.0E-5) using Blast2GO (http://www.blast2go.com, Conesa et al., 2005; RRID: SCR_005828). The unigenes were further classified using GO Slim (http://www.geneontology.org/GO.slims.shtml). To assign the detected unigenes to biological pathways, Kyoto Encyclopedia of Genes and Genomes (KEGG) pathway annotation was conducted using the online KEGG Automatic Annotation Server (KAAS, http://www.genome.jp/kegg/kaas/). The DEGs were analyzed for GO category enrichment and KEGG pathway enrichment using AgriGO (Du et al., 2010; RRID: SCR_006989) and KAAS, respectively, using Fisher's Exact Test and FDR correction.

### Validation of RNA-Seq data by qRT-PCR

To validate the gene expression profiles identified by RNA-Seq, 1 μg of RNA was reverse transcribed in a 20-μL volume with PrimeScript RT Master Mix (TaKaRa), according to the manufacturer's instructions. Gene-specific primers were designed with Geneious Pro 4.8.5 to be between 18 and 27 bp long, with a GC content of 40–60%, a melting temperature (Tm) of 57–63°C, and a PCR product range of 160–300 bp. For genes that have been sequenced previously, the full-length sequences were used to design primers. Primers used in this study are listed in Table S1. QRT-PCR was performed with SsoAdvanced PreAmp Supermix (Bio-Rad Laboratories, Hercules, CA, USA) in a Bio-Rad CFX96 Touch PCR Detection System with the following conditions: 95°C for 10 min and then 45 cycles of 95°C for 15 s and 58–66°C for 30 s, followed by a melt cycle of 65°C for 5 s and 95°C for 15 s. Reactions were performed in triplicate, with a negative nuclease-free water control in each run. Sweet potato H2B and UBI encoding genes were used as a double internal control for normalization of the gene expression data (Park et al., 2012). The specificity of qRT-PCR products was confirmed by performing a melting temperature analysis and agarose gel electrophoresis detection followed by sequencing. The relative expression levels of the genes of interest were quantified with the delta threshold cycle (ΔCt) method (Schmittgen and Livak, 2008), referenced to the internal control. The experiments were repeated three times in independent qRT-PCR reactions.

Pearson's correlation analysis of gene expression and trait values was performed with SPSS (RRID: SCR_002865) version 20.0, and tests of significance were two-sided.

## Results

### Dry matter and starch accumulation during SR development

Three sweet potato varieties, YS33, XS22, and SQ52-7, with relatively high, medium, and low starch contents, were respectively selected based on the trait values measured. At the harvest stage (150–170 DAP, depending on the climate and observed growth recorded in each year), the mean dry matters of the three genotypes over the 3 years (2011–2013) were 34.469 ± 2.385%, 27.957 ± 1.137%, and 13.725 ± 1.473%, respectively, while the corresponding average starch contents were 23.623 ± 2.073%, 17.961 ± 0.989%, and 5.588 ± 1.280%, respectively (Table S2).

**Figure 1:**
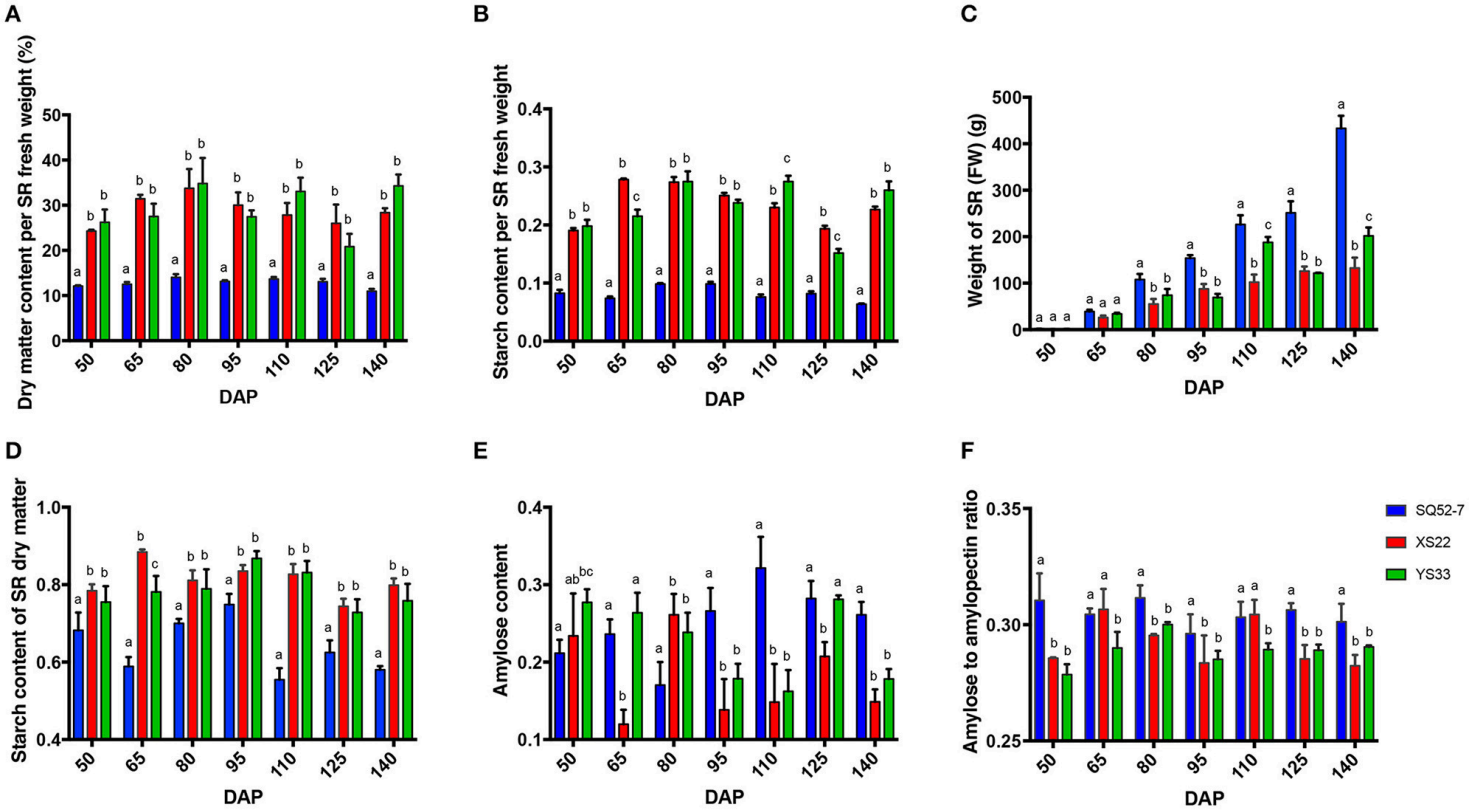
Dynamic changes of dry matter, starch properties, and SR weight during SR development. Dynamic changes of **(A)** dry matter content per SR fresh weight, **(B)** starch content per SR fresh weight, **(C)** SR fresh weight, **(D)** starch content of SR dry matter, **(E)** amylose content, and **(F)** amylose to amylopectin ratio in the SR starch during SR development in the SQ52-7, XS22, and YS33 genotypes. Error bars indicate the standard deviation from three independent replicates.

To compare the dry matter and starch accumulation in sweet potato SRs, the starch-related traits of the underground roots harvested at regular intervals from 50 to 140 DAP were measured. The dry matter and starch content of flesh fluctuated during SR development (Figures 1A,B). During SR development, the fresh weight of all of the genotypes accumulated significantly (*P* < 0.0001; Figure 1C and Table S3). Dry matter accumulates during SR development, but the ratio of dry matter to the total fresh weight of the SR was not always enhanced. The dry matter content of SQ52-7 was significantly lower than that of YS33 and XS22 (both *P* < 0.0001; Figure 1A and Table S4), indicating that the SRs of SQ52-7 contained more water than did those of the other two varieties. Moreover, the dry matter content of SQ52-7 did not significantly increase from 50 to 140 DAP (from 10.978 ± 0.080% to 14.050 ± 0.127%, *P* = 0.286; Figure 1A), but the fresh weight of its SRs increased more rapidly from 50 to 140 DAP (from 2 to 422 g, Figure 1C) than that of YS33 (from 1 to 217 g) and XS22 (from 1 to 132 g), especially from 125 to 140 DAP (increased from 280 to 422 g in the case of SQ52-7), suggesting that the increase in dry matter was not the main factor contributing to the rapid increase of SR fresh weight in SQ52-7.

The starch content of the total dry matter in SQ52-7, XS22, and YS33 ranged from 55.472 to 74.885%, 74.436 to 88.458%, and 72.829 to 86.795%, respectively, and peaked at 95 DAP (Figure 1D). SQ52-7 SRs contained significantly lower starch contents in the dry matter than did YS33 and XS22 SRs (*P* < 0.05 at all 7 stages examined; Figure 1D and Table S4). We found that the total starch in the dry matter of SRs of XS22 was higher than that of YS33 at most developmental stages (Figure 1D), indicating that XS22 accumulates higher levels of starch in its SR dry matter than does YS33. However, the dry matter content of XS22 SRs was lower than that of YS33 at 110 and 140 DAP (Figure 1A) and significantly lower than that of YS33 at harvest (Table S2, *P* < 0.0001), indicating that YS33 SRs might accumulate higher levels of dry matter components in addition to starch than XS22 SRs.

The starch composition changed during SR development in the three tested varieties (Figures 1E,F). Although SQ52-7 had the lowest starch content of the three varieties, it had the highest amylose content and amylose to amylopectin ratio. XS22 had the lowest amylose to amylopectin ratio among the three varieties.

### Sugar composition of the SR dry matter of the three sweet potato varieties

**Figure 2:**
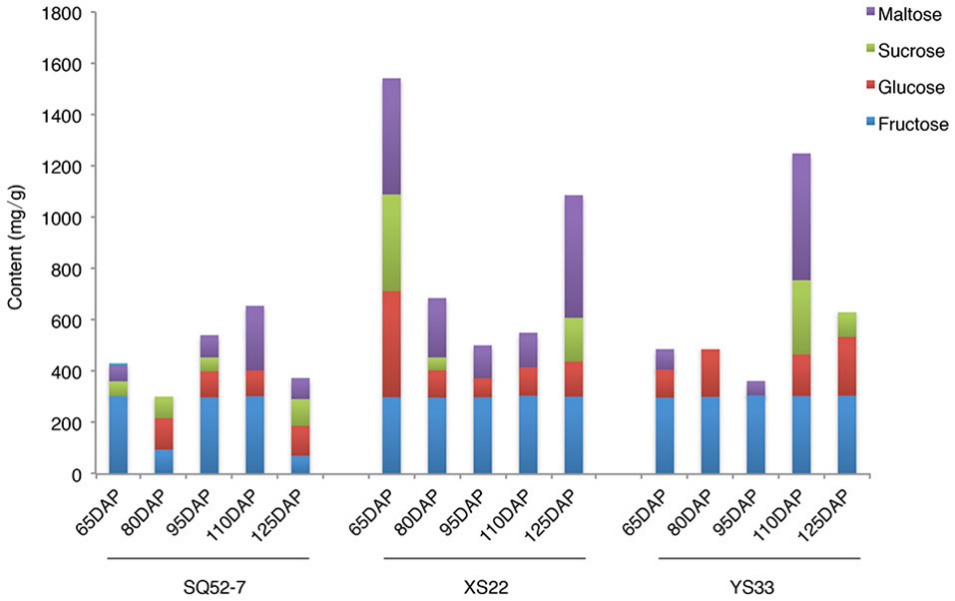
Dynamic changes of sugar content during SR development.

We measured the content of various sugars, including sucrose, fructose, glucose, and maltose, in the dry matter of the 15 SRs using HPLC. There was no detectable sucrose accumulation in the SRs of YS33 at 65, 80, and 95 DAP (Figure 2). In XS22, sucrose was only present in the SR dry matter in the early and late stages of SR development, whereas the SRs of SQ52-7 contained sucrose during four of the five developmental stages examined. YS33 and XS22 exhibited more fructose and glucose in the SR dry matter than did SQ52-7. The SRs of XS22 contained high levels of maltose, possibly due to the high starch content in the dry matter of this genotype.

### Transcriptome assembly and functional annotation

A total of 8.35E + 09 high-quality reads were generated with an average trim read length of 98.47 bp. For each sample, from 5.27E + 07 to 6.14E + 07 raw reads were obtained, and the total number of bases ranged from 5.33E + 08 to 6.21E + 08. After trimming the adapter sequences and removing low quality sequences, approximately 7.91E + 09 sequences from all 15 samples were assembled into 241,386 transcripts and 112,336 unigenes (≥ 200 bp) using Trinity. The average length of the transcripts and unigenes was 973.89 and 662.39 bases, respectively (Table S5). The unigene length distribution is illustrated in Figure S1. The sequencing data were deposited in the NCBI Sequence Read Archive (SRA, http://www.ncbi.nlm.nih.gov/Traces/sra) under accession number SRP075405.

**Figure 3:**
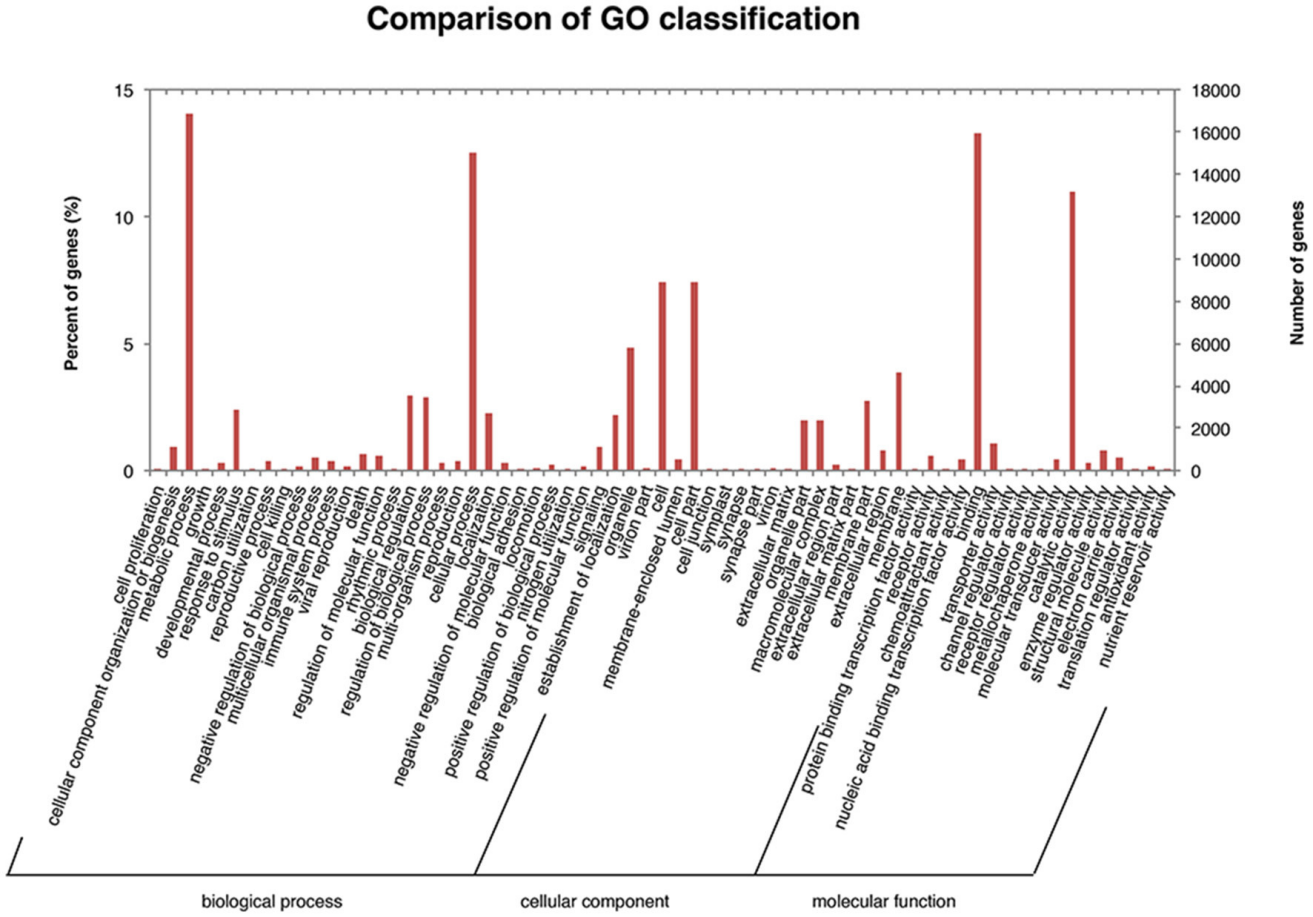
Gene ontology classification of assembled unigenes.

The unigenes were annotated using the NCBI Nr, Swiss-Prot, TrEMBL, CDD, Pfam, and KOG databases. In this analysis, all unigenes exhibited high sequence similarity (>30%) with known gene sequences at a cut-off *E*-value of ≤ 1.0E-5 (Table S6), indicating a high level of similarity between our sequences and those in the BLAST database. Based on the GO annotation, 10498 unigenes were grouped into three functional GO categories, i.e., Biological Process (BP; 51370 sequences), Molecular Function (MF; 35956), and Cellular Component (CC; 32308), with subsets of sequences further divided into 30, 18, and 17 subcategories in these three groups, respectively (Figure 3). There was a high representation of “metabolic process” and “cellular process” in the category BP, which included 14.052% and 12.487% of the sequences in these subcategories, respectively. Furthermore, there was an enrichment of “binding” (13.308%) and “catalytic activity” (11.013%) in the MF parental category, and a high representation of “cell” (7.419%), “cell part” (7.419%), and “organelle” (4.819%) in the CC category.

To assign the detected unigenes to biological pathways, all of the unigenes were compared against the KEGG pathways database using BLASTX with an *E*-value cutoff of < 1.0E-5. We mapped 11873 unigenes to 321 KEGG pathways, and the highly-represented pathways included Ribosome (ko03010, 629 unigenes), Protein processing in endoplasmic reticulum (ko04141, 350 unigenes), Plant-pathogen interaction (ko04626, 336 unigenes), Plant hormone signal transduction (ko04075, 336 unigenes), and Starch and sucrose metabolism (ko00500, 330 unigenes).

### Expression patterns of unigenes in 15 SR samples

**Figure 4:**
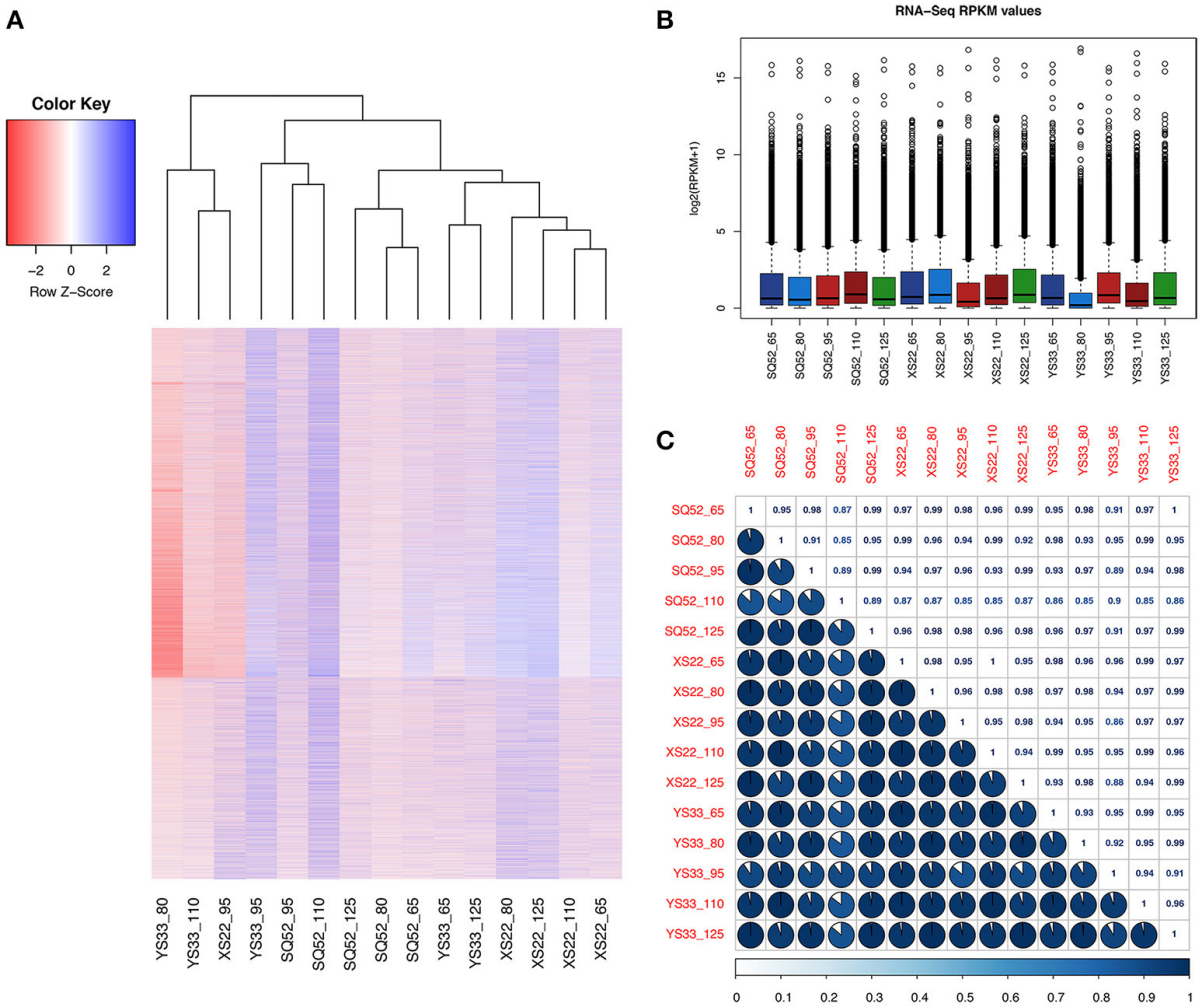
Comparison of the transcriptomes among the tested SR samples. **(A)** Heatmap plotting and cluster analysis of 15 SR databases based on the expression pattern and abundance of unigenes; **(B)** Box plot showing the RPKM distribution of unigenes in the 15 samples; **(C)** Correlation analysis among samples. The correlation matrix was performed by comparing the values of the whole transcriptome in 15 samples, and Pearson's correlation coefficient among samples was analyzed using R scripts.

We found that the examined SR samples exhibited different gene expression patterns (Figure 4). The transcript number in the 15 SR samples ranged from 49916 (in SRs of YS33 at 80 DAP) to 80330 (in SRs of YS33 at 95 DAP, Table S7). The expression patterns of unigenes in SQ52-7 SRs at 110 DAP was different from that of other SR samples (Figure 4). For each genotype, the unigene expression patterns during the early developmental stages (65 DAP or 80 DAP) were similar to those during the late developmental stages (110 DAP or 125 DAP). The 95 DAP samples from all three genotypes had unique unigene expression patterns that distinguished them from the other SR samples (Figure 4). A similar unigene expression pattern was exhibited by YS33 at 110 DAP, XS22 at 95 DAP, and YS33 at 80 DAP, that had fewer expressed transcripts but higher max unigenes expression abundance when compared to another samples (Figure 4 and Table S7). The correlation coefficient (*r*^2^) of transcriptomes among SRs ranged from 0.85 to 1 (Figure 4C), indicating that the 15 SR samples share relatively similar expression patterns, and ensuring the reliability of sequencing and sampling indirectly.

### Genotypic and developmental stage-specific unigenes

**Figure 5:**
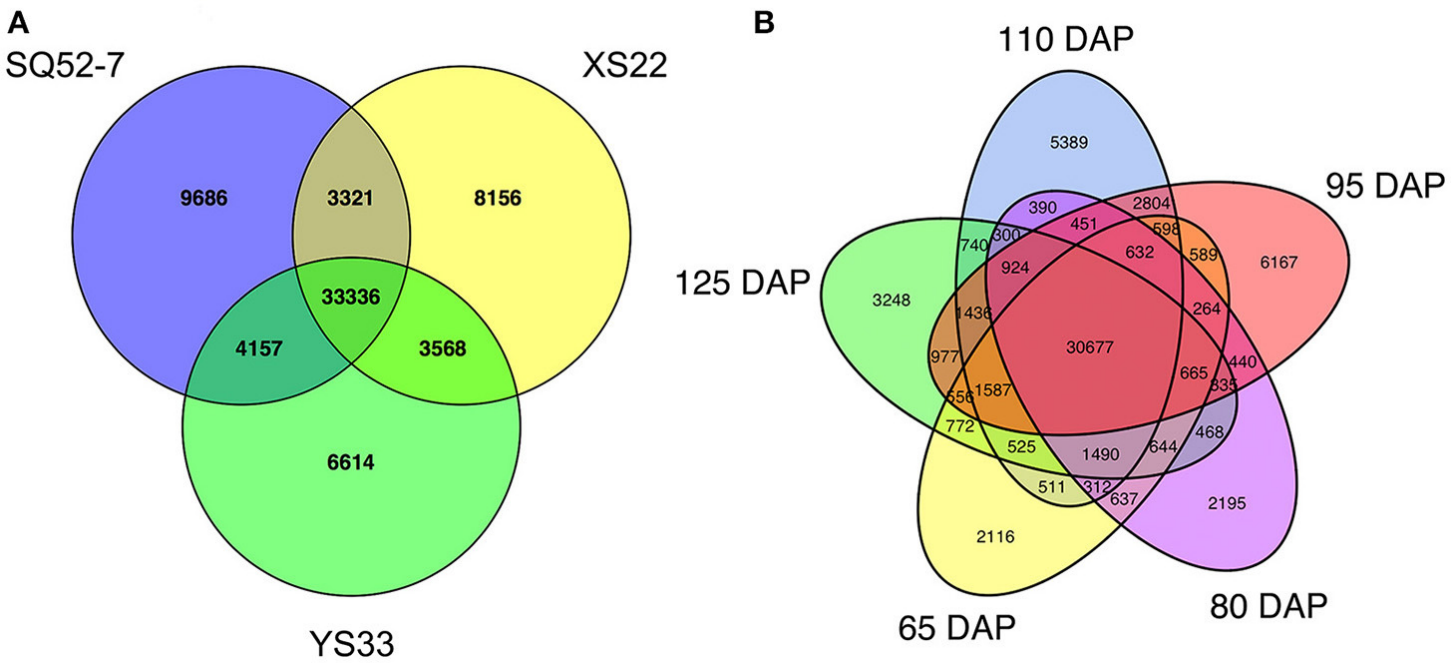
Expressed unigenes identified in the 15 sweet potato SRs. Venn diagrams were generated to identify sweet potato **(A)** genotype- and **(B)** developmental stage-specific expressed unigenes and common expressed unigenes.

After excluding unigenes with undetected expression (RPKM < 0.5), 19115 and 24456 unigenes specifically expressed in one developmental stage and one genotype were identified, respectively. Genotype-specific unigenes accounted for 35.5% of the total number of expressed unigenes, which is approaching the percentage of commonly expressed unigenes in the sweet potato genotype (48.4%), indicating that transcriptome variation was greater among genotypes than among developmental stages (Figure 5). Several starch and sucrose metabolism-related genes exhibited genotype-specific expression. An α-amylase encoding unigene (comp26344_c0_seq1) and an invertase inhibitor (INVinh) encoding unigene (comp67966_c0_seq3) were only detected in SRs from SQ52-7, and two α-amylase inhibitor encoding unigenes were only detected in SQ52-7 (comp109727_c0_seq1 and comp90515_c0_seq1) and YS33 (comp108417_c0_seq1 and comp108272_c0_seq1), respectively. Two *SuSy* genes (comp2257_c0_seq1 and comp26077_c0_seq1) and a β*-amylase* (*BMY*) gene (comp94608_c0_seq1) were only expressed in XS22 SRs, and three transporters, including a glucose transporter encoding unigene (comp104813_c0_seq1) and two glucose-6-phosphate/phosphate translocator (G6PPT) encoding unigenes (comp32901_c0_seq1 and comp33012_c0_seq1), were only expressed in XS22 SRs. We found that the AGPase large subunit 4 encoding unigene *IbAGPb3* was only expressed in YS33 SRs at 95 DAP.

### DEGs involved in starch and sucrose metabolism

To investigate the mechanism underlying starch accumulation in SR, and identify the regulators that contribute to variation in starch properties among sweet potato genotypes, we identified the DEGs involved in starch and sucrose metabolism among the 15 SR samples. These unigenes mainly include genes encoding key enzymes involved in starch biosynthesis and degradation, sucrose metabolism, interconversion between sugar and starch, and transporters (Table S8, Figure S2 and Data S1).

**Figure 6:**
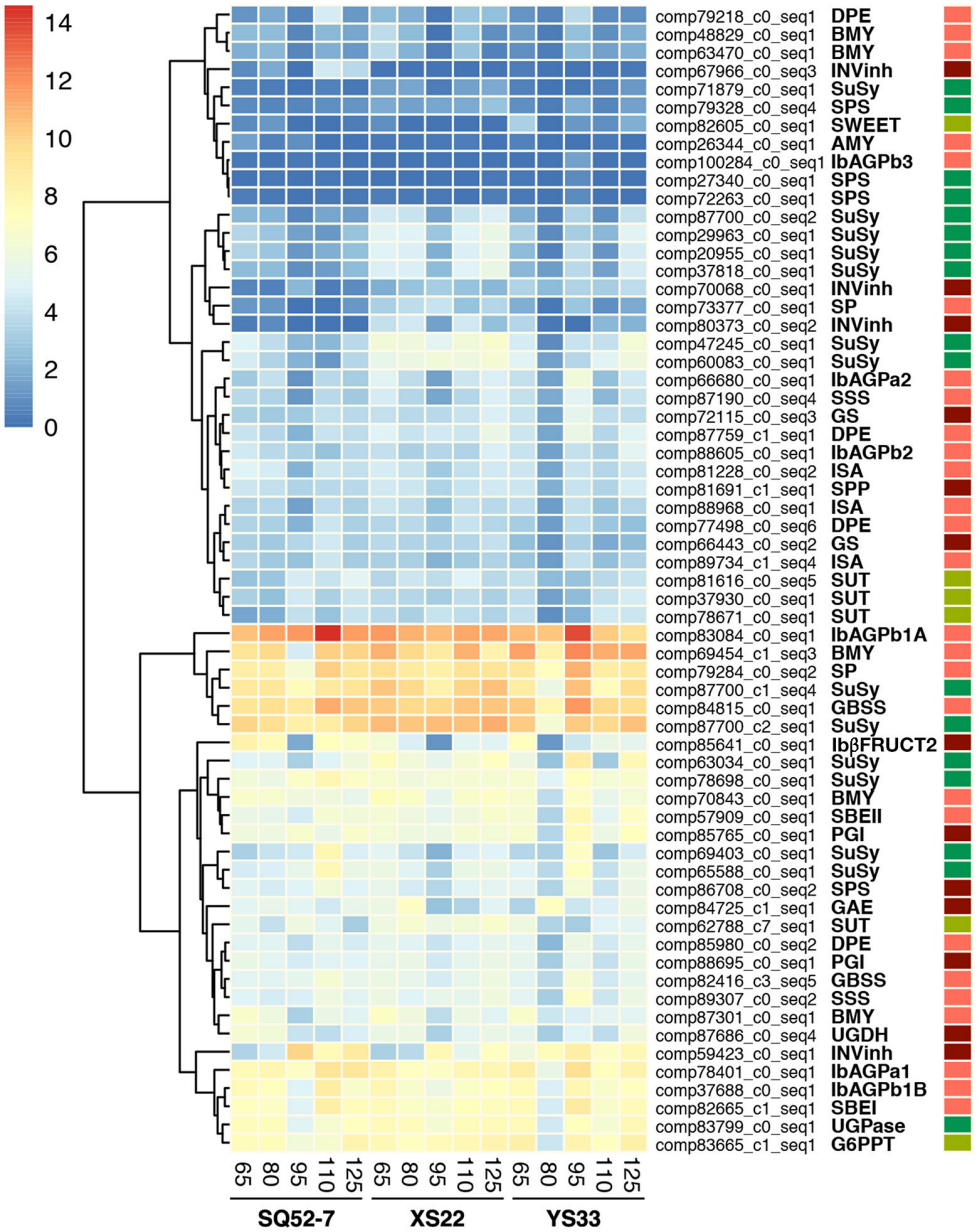
Expression patterns of unigenes encoding enzymes involved in starch and sucrose metabolism during SR development in three sweet potato genotypes. Cluster analysis was performed to group DEGs with similar expression levels and patterns based on the normalized log2-transformed RPKM values of DEGs. The abbreviations of enzymes and transporters encoded by DEGs are shown in Figure S2. GS, glycogen (starch) synthase (EC 2.4.1.11); IbAGPa1, IbAGPa2, IbAGPb1A, IbAGPb1B, IbAGPb2, ADP-glucose pyrophosphorylase (AGPase, EC 2.7.7.27) small subunit 1, 2, and large subunit 1, 2, and 3, respectively; IbβFRUCT2, β-fructofuranosidase (EC 3.2.1.26); INVInh, invertase inhibitor; SBEI and SBEII, class I and II starch branching enzyme (EC 2.4.1.18), respectively. Red, dark red, green, and pale green rectangles on the right side indicate DEGs involved in starch granule formation and degradation, sucrose metabolism, sucrose synthesis and conversion, and DEGs encoding transporters, respectively.

Cluster analysis assigned these DEGs into two groups (Figure 6), based on their expression levels (high and low expression unigenes). Among the high expression group, several unigenes showed high expression throughout SR development in all three genotypes, such as the AGPase large subunit 1 encoding gene *IbAGPb1A* (comp83084_c0_seq1), the GBSS encoding gene *IbGBSSI* (comp84815_c0_seq1; Kimura et al., 2000), the SP encoding gene *IbSP* (comp79284_c0_seq2; Lin et al., 1991), two SuSy encoding genes (unigene comp87700_c1_seq4, which have partial sequence similarity with the reported Yusu 303 SuSy mRNA, and unigene comp87700_c2_seq1), and one BMY encoding gene (comp69454_c1_seq3; Yoshida and Nakamura, 1991). Among these unigenes, four (all except *IbAGPb1A* and *IbGBSSI*) showed higher expression in YS33 and XS22 than in SQ52-7 at most developmental stages. In addition, genes (comp83799_c0_seq1, comp82665_c1_seq1, comp59423_c0_seq1) encoding UDP-glucose pyrophosphorylase (UGPase), SBEI, and INVinh, respectively, and the G6PPT transporter encoding gene (comp83665_c1_seq1) also exhibited high expression in these SR samples. Among the low expression group, some unigenes were expressed at low levels throughout SR development in the three genotypes investigated, such as unigenes (comp27340_c0_seq1, comp72263_c0_seq1, and comp79328_c0_seq4) encoding sucrose-phosphate synthase (SPS), two BMY encoding genes (comp48829_c0_seq1 and comp63470_c0_seq1), and a disproportionating enzyme (DPE) encoding gene (comp79218_c0_seq1).

We also found that most DEGs involved in starch and sucrose metabolism were highly expressed at 95 DAP in the three genotypes, suggesting that 95 DAP might be a critical stage in sucrose metabolism or starch biosynthesis in sweet potato SRs.

### Expression patterns of genes involved in starch metabolism

Unigenes encoding the five key enzymes [AGPase, GBSS, SSS, SBE, and starch de-branching enzyme (DBE, α-1, 6-glucanohydrolase)] involved in starch granule formation (Figure S2) showed different expression patterns in the SRs of the three genotypes (Figure S3). *IbAGPb3* showed weak expression in YS33 at 95 DAP (RPKM > 0.5), and was not expressed in the other samples (RPKM < 0.5). QRT-PCR analysis indicated that this gene was expressed at higher levels in XS22 and YS33 at 95 DAP than in other samples, but its expression level was very low compared with that of the other AGPase encoding genes (Figure S3D).

*IbISBEI, IbISBEII* and two *IbGBSS* genes showed high expression throughout SR development in all three genotypes. Two *SSS* genes, which were not previously reported in sweet potato, also showed relatively high expression in the SRs of all three genotypes. Expression of a starch synthase III encoding gene (comp89307_c0_seq2) was higher than that of starch synthase I encoding gene (comp87190_c0_seq4) in the three genotypes, but both of these genes were expressed at higher levels in YS33 or XS22 than in SQ52-7 (Figure 6 and Figure S3). Two types of DBE exist in plants, limit dextrinase (also called pullulanase) and ISA. In this study, only three ISA encoding DEGs were detected, and they were all expressed at relatively low levels (RPKM from 1.53 to 28.45) in the 15 SR samples.

Starch phosphorylase (SP) was reported to be involved in starch granule formation (Zeeman et al., 2010; Schreiber et al., 2014), catalyzing the extension of glucan chains using glucose-1-phosphate as a substrate or releasing glucose-1-phosphate from glucan chains during starch granule formation. Two SP encoding genes (comp79284_c0_seq2 and comp73377_c0_seq1) were differentially expressed during SR development (Figure 6 and Figure S3), and they were both expressed at higher levels in XS22 and YS33 than in SQ52-7. Two glycogen synthase (GS) encoding genes exhibited different expression patterns during SR development in the three genotypes (Figure 6 and Figure S4). These genes showed 81 and 78% sequence identity with starch synthase genes in tobacco (*Nicotiana tomentosiformis*), implying that these two genes might be novel starch synthase encoding genes in sweet potato.

Granule-bound starch synthases (GBSS) catalyzes amylose biosynthesis, and SSS and SBE are essential for amylopectin biosynthesis in higher plants (Zhang et al., 2008; Zeeman et al., 2010). In SQ52-7, the amylose to amylopectin ratio in SRs was higher than that in YS33, while XS22 had the lowest ratio of the three genotypes (Figures 1E,F). Two *GBSS* genes were expressed at higher levels in the SRs of YS33 and SQ52-7 than in those of XS22 (Figure 6, Figures S3G,H), indicating that the expression of *IbGBSS* may affect the amylose content in the SRs. However, there was no significant correlation between the expression level of the two detected *IbGBSS* genes, which was quantified by qRT-PCR, and amylose content or amylose to amylopectin ratio in these SRs. Two *IbSSS* genes showed higher expression in YS33 than in XS22 and SQ52-7 (Figure 6, Figures S3I,J). The expression levels of the *IbSSS* unigene comp87190_c0_seq4 were significantly negatively correlated with the amylose to amylopectin ratio (*r*^2^ was -0.554, with a *P*-value of 0.032). DBE was involved in amylopectin synthesis (Zeeman et al., 2010). *IbSBEI, IbSBEII*, and three *IbISA* genes were not differentially expressed among the three genotypes (Figure 6 and Figure S5), and the expression of these genes was not correlated with the amylose content or amylose to amylopectin ratio in these SRs, indicating that the expression of genes encoding SBE and ISA during starch granule formation did not directly contribute to the amylose to amylopectin ratio.

Starch could be degraded either directly by amylase or via the phosphorylation pathway. In this study, we identified five unigenes encoding β-amylase (BMY). Three of these genes exhibited high levels of expression in the 15 samples (Figure 6 and Figure S6). Unigene comp69454_c1_seq3 was expressed at higher levels in YS33 than in XS22, and was expressed at lowest level in SQ52-7 (Figure 6 and Figure S6A). SP catalyzes starch degradation via the reversible phosphorylation of α-glucan. We identified two SP encoding DEGs. One of them (comp79284_c0_seq2) was expressed at high levels in all three genotypes, particularly in YS33 at 95 DAP (Figure 6 and Figure S3K). Expression of β*-amylase* and *SP* genes indicates that both starch degradation pathways exist in sweet potato SRs. Indeed, β-amylase contributes to the characteristic sweet taste of sweet potato SRs (Anwar et al., 2009). Except for the α*-amylase* unigene specifically expressed in SQ52-7, no other α-amylase encoding DEG was identified in our analysis. Disproportionating enzyme (DPE) also participates in starch degradation and biosynthesis (Ohdan et al., 2005; Zeeman et al., 2010). We identified four differentially expressed *DPE* genes, which were strongly expressed in the SRs of all three genotypes at 95 or 110 DAP (Figure 6 and Figure S7).

### Gene expression pattern of enzymes related to the interconversion of sucrose and starch

Sucrose synthase (SuSy) catalyzes the reversible conversion of UDP-glucose and fructose into sucrose, but mainly acts to decompose sucrose into UDP-glucose and fructose to provide substrate for starch synthesis (Table S8 and Figure S2, Schreiber et al., 2014). We detected a total of 13 SuSy encoding DEGs with diverse expression patterns. The unigene comp71879_c0_seq1 was expressed at low levels, but all of the other 12 SuSy genes showed high expression in the detected SRs. Two *SuSy* genes (comp87700_c1_seq4 and comp87700_c2_seq1) showed extremely high levels of expression during SR development in the three genotypes. Four *SuSy* genes (comp87700_c0_seq2, comp29963_c0_seq1, comp20955_c0_seq1, and comp37818_c0_seq1) showed similar expression patterns; however, it remains to be determined whether these unigenes represent the same *SuSy* gene (Figure 6 and Figure S8). UDP-glucose pyrophosphorylase (UGPase) is a key enzyme in the starch and sucrose biosynthesis pathway (Figure S2). Expression detection results demonstrated that the UGPase encoding DEG (comp83799_c0_seq1) was expressed at higher levels in YS33 and XS22 than in SQ52-7 (Figure 6 and Figure S9). Correlation analysis showed that *SuSy* (comp87700_c1_seq4) and *UGPase* (comp83799_c0_seq1) expression were positively correlated with starch content in the dry matter (*r*^2^ were 0.476 and 0.479, with *P*-values of 0.073 and 0.071, respectively) in the three genotypes, indicating their critical roles in SR starch accumulation in sweet potato.

### Genes involved in sucrose metabolism

Sucrose synthesis involves SPS, which converts UDP-glucose and fructose-6-phosphate into sucrose-6-phosphate; sucrose-phosphate phosphatase (SPP), which converts sucrose-6-phosphate into sucrose (Wind et al., 2010); and glucose-6-phosphate isomerase (PGI), which catalyzes the interconversion of glucose-6-phosphate and fructose-6-phosphate. SPS, SPP, PGI, SuSy, and UGPase all directly or indirectly participate in starch biosynthesis through interconverting sugar and UDP-glucose (Table S8 and Figure S2, Ferreira and Sonnewald, 2012). These enzymes all catalyze reversible reactions. Our analyses showed that DEGs encoding SPS, SPP, and PGI were differentially expressed during SR development, but only one SPS (comp86708_c0_seq2) and two PGI encoding unigenes (comp85765_c0_seq1 and comp88695_c0_seq1) were highly expressed in SRs. There was no direct correlation between their expression levels and the starch and sugar properties of the three genotypes examined (Figure 6 and Figure S10).

UDP-glucose 6-dehydrogenase (UGDH) (comp87686_c0_seq4) and UDP-glucuronate 4-epimerase (GAE) encoding genes (comp84725_c1_seq1) showed differential expression during development, but not among genotypes (Figure 6 and Figure S11). The two enzymes catalyze the conversion of UDP-glucose to products that participate in other metabolic pathways, such as pentose and glucuronate interconversions, ascorbate and aldarate metabolism, and nucleotide sugar metabolism (Figure S2, Ferreira and Sonnewald, 2012; Lai et al., 2014).

**Figure 7:**
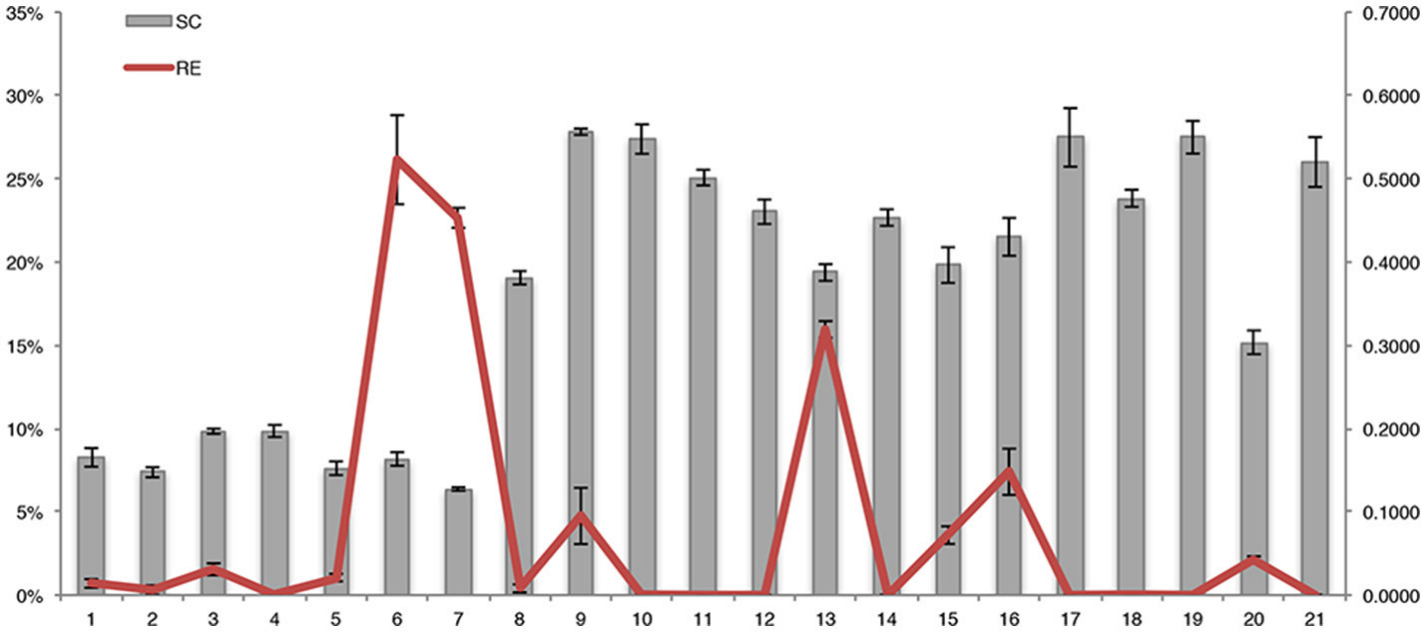
Dynamic changes in the starch content and the expression pattern of *Ibβfruct2* during SR development in the three sweet potato varieties. SC, starch content per SR fresh weight; RE, relative expression (ΔCt) of *Ibβfruct2*.

An invertase encoding gene, *Ibβfruct2* (comp85641_c0_seq1), showed differential expression in the SRs of the three genotypes. Expression analysis demonstrated that this gene had higher expression in SQ52-7 than in XS22, and lowest expression in YS33 (Figures 6, 7), which was opposite to the tendency for SR starch accumulation (Figure 7). *Ibβfruct2* encodes the vacuolar acid invertase IbβFRUCT2, which catalyzes the conversion of sucrose to fructose and glucose in the vacuole. The expression pattern of *Ibβfruct2* and the starch content of SRs revealed that there is a significant negative relationship between the expression of *Ibβfruct2* and the starch content of SRs at 80, 110, and 140 DAP (*r*^2^ were -0.999, -0.850, and -0.884, with *P*-values of 0.015, 0.008, and 0.031, respectively). Invertase activity is regulated by the invertase inhibitor (INVinh) (Rausch and Greiner, 2004). Four INVinh encoding unigenes (comp59423_c0_seq1, comp70068_c0_seq1, comp80373_c0_seq2 and comp67966_c0_seq3) were differentially expressed among the SR samples from different genotypes or developmental stages (Figure 6 and Figure S12). Among them, comp70068_c0_seq1 showed the highest expression in YS33 and lowest expression in SQ52-7 (Figure 6 and Figure S12A), which is consistent with the tendency for SR starch content. Besides, a positive correlation between the expression of this unigene and the starch content of SRs during development (*r*^2^ was 0.839–0.960) was also observed. Expression of the *INVinh* unigene comp70068_c0_seq1 was negatively correlated with that of *Ibβfruct2* (*r*^2^ was -0.601 – −0.975), and the expression of another *INVinh* unigene (comp80373_c0_seq2) also showed a significant negative correlation with that of *Ibβfruct2* (−0.689 – −1.000, *P* = 0.010 at 65 DAP). However, INVinh has been reported to mediate the post-translational regulation of invertase, and the activity of one invertase might be regulated by several INVinhs (Link et al., 2004; Lin et al., 2013). Therefore, it remains to be determined if the two *INVinh* unigenes encode inhibitors of IbβFRUCT2 at the protein expression level.

### DEGs participate in water metabolism in sweet potato

**Figure 8:**
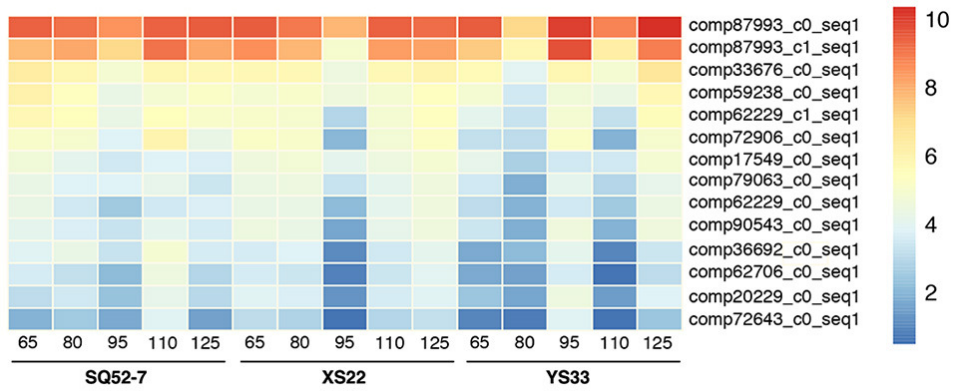
Heatmap showing expression patterns of pyrophosphate-energized vacuolar membrane proton pump encoding unigenes. Normalized log2-transformed RPKM gene expression values were used to plot the heatmap.

To identify genes controlling the SR water content, we analyzed water metabolism-related DEGs, including genes involved in the GO terms vacuole, vacuolar membrane, and water transport, and genes responsive to water deprivation and osmotic stress, in our SR transcriptomes. We detected two kinds of DEGs, one of which encodes a pyrophosphate-energized vacuolar membrane proton pump (vacuolar H^+^-PPase) and the other of which encodes a probable aquaporin. We detected DEGs encoding three groups of aquaporin: the plasma membrane intrinsic protein (PIP), tonoplast intrinsic protein (TIP), and Nodulin 26-like intrinsic membrane protein (NIP). Although none of the aquaporin unigenes showed differential expression among the SRs of the three genotypes examined, they did exhibit dynamic changes in gene expression during SR development (Figure S13). Vacuolar H^+^-PPase unigenes showed higher expression in the SRs of SQ52-7 than in those of XS22 and YS33, particularly in the case of XS22 at 95 DAP, and YS33 at 65, 80, and 110 DAP (Figure 8). Vacuolar H^+^-PPase is abundant and ubiquitous in the vacuolar membranes of plant cells, and is instrumental in the transport, sugar storage, and osmoregulatory functions of the vacuole (Fuglsang et al., 2011). The differential expression of these unigenes among the three genotypes examined indicated the roles of the vacuole and water metabolism in regulating SR starch content in sweet potato.

## Discussion

### Both water content and starch accumulation in dry matter affect the starch content of SRs

Starch quality affects the potential uses of sweet potato in starch-based industries and biofuel ethanol production, and the dry matter content, starch content, and starch composition are therefore traits of high agronomic importance in sweet potato. However, the starch and sugar content of storage organs are complex traits controlled by multiple genetic and environmental factors (Li et al., 2008; Schreiber et al., 2014). In our previous studies, we found that the SR dry matter and starch content varied greatly among genotypes, but that for each genotype, these traits were relatively stable among different planting years or environments, and the genotype had a larger effect than did the environment on these traits (Lu et al., 2015; Zhang et al., 2016). These findings emphasize that the dry matter and starch content of sweet potato SRs is mainly controlled by genetic factors. One aim of our current study was to identify the factors that determine the variation of dry matter and starch content among different sweet potato genotypes.

Sweet potato SRs contain both water and dry matter. The dry matter of sweet potato SRs consists of sugar, starch, fat, and other minor components, but starch accounts for the largest portion (50–80%) of the dry matter in SRs (Rukundo et al., 2013; Zhou et al., 2015). Our results showed that compared with XS22 and YS33, the SRs of SQ52-7 had a lower dry matter content, and thus a higher water content. We found that the dry matter content of SRs in SQ52-7 did not increase rapidly, even though the fresh weight of SRs increased rapidly during SR development (Figure 1C) compared to the other two varieties, possibly due to the rapid accumulation of water in this organ. Moreover, the dry matter starch content of SQ52-7 SRs is significantly lower than that of XS22 and YS33, and the low dry matter content and low starch accumulation in SR dry matter underlie the very low SR starch content in SQ52-7.

XS22 contained a lower SR starch content at the harvest stage (17.961 ± 0.989%) than did YS33 (23.623 ± 2.073%), and the dry matter content of XS22 SRs (27.957 ± 1.137%) was also significantly lower than that of YS33 SRs (34.469 ± 2.385%) (*P* < 0.0001), but we found that starch accumulation in the dry matter of XS22 SRs was higher than that in YS33 SRs at most detected developmental stages. The starch and sugar contents in the dry matter of SRs in XS22 were both higher than those in YS33 at most developmental stages. Compared to YS33, the lower starch content per SR fresh weight detected in XS22 was mainly due to the lower dry matter content and higher water content in the SR at the harvest stage, and not due to starch accumulation in the dry matter. Thus, the dry matter or water content of SR flesh, combined with the starch accumulation in the dry matter, determine the starch content of SRs, and differences in water content and starch accumulation in the dry matter of SRs also contribute to differences in SR starch contents among genotypes.

### Starch granule formation and degradation were not the decisive factors underlying SR starch content variation

To decipher the molecular mechanism underlying starch accumulation in SRs, we detected DEGs encoding key enzymes involved in starch granule formation. Surprisingly, these genes were not always expressed at much lower levels in the SRs of SQ52-7, which have a low SR starch content and starch accumulation in the dry matter, than in those of YS33, which have a high starch content and starch accumulation in the dry matter. Furthermore, there was not a direct correlation between the expression of genes encoding any of the enzymes involved in starch degradation, including SP, DPE, and BMY, and starch content traits in the SRs of the three genotypes. These results indicate that the expression of genes encoding starch granule-synthesizing or degradation enzymes are not the decisive factors contributing to the SR starch content variation among sweet potato genotypes.

AGPase catalyzes the formation of ADP-glucose, the substrate for starch synthesis, and thus constitutes the first, rate-limiting step in starch biosynthesis (Hannah and James, 2008; Geigenberger, 2011). However, in our RNA database, only *IbAGPb1A, IbAGPb1B*, and *IbAGPa1*, which encode the AGPase large subunit 1A and 1B and small subunit 1, respectively, were markedly expressed in developing SRs, and *IbAGPb2, IbAGPb3*, and *IbAGPa2*, which encode the AGPase large subunit 3 and 4, and small subunit 2, respectively, were expressed at low levels compared with those of the other three AGPase subunit encoding genes. Especially *IbAGPb3*, which was scarcely expressed in sweet potato SRs. This result is similar to the findings of a previous study that examined the expression of genes encoding AGPase subunits in sweet potato (Zhou et al., 2016), which showed that *IbAGPb3* was only expressed in leaves and *IbAGPb2* was expressed in sink tissues at low levels. However, our results also showed that *IbAGPb3* was expressed at 95 DAP in the SRs of YS33 and XS22 (Figure 6 and Figure S3D); thus, the tissue specificity and role of this subunit remain to be determined.

Interestingly, the expression of most DEGs encoding key enzymes involved in starch and sucrose metabolism, such as AGPase, GBSS, SSS, SP, and SuSy, peaked at 95 DAP. This result is in agreement with our finding that starch accumulation in the dry matter of SRs of the three genotypes examined all peaked at 95 DAP, indicating that 95 DAP is an important time point in starch biosynthesis. This developmental stage should be the focus of future studies of SR starch traits of sweet potato.

### Gene expression of starch-synthesizing enzymes is not directly correlated with starch composition in SRs

The amylose content and amylose to amylopectin ratio are important factors affecting starch structure and properties. In sweet potato, amylose-free and high-amylose transgenic sweet potato plants have been produced by inhibiting the expression of genes encoding sweet potato GBSSI and SBEII through RNA interference, respectively (Shimada et al., 2006; Kitahara et al., 2007; Otani et al., 2007). These results showed the critical role of these enzymes in controlling starch composition. In our RNA-seq analysis, the two GBSS encoding genes were expressed at relatively higher and lower levels in the SRs of genotypes with higher and lower amylose to amylopectin ratios, respectively, and the expression of SSS encoding gene was significantly correlated with the amylose to amylopectin ratio, indicating that variation in the expression of these GBSS and SSS genes might directly influence the variation in starch composition among different sweet potato genotypes. However, the expression of genes encoding other starch-synthesizing enzymes, including AGPase, SBEI, SBEII, and ISA, showed no direct correlation with the starch composition of SRs of the three genotypes. These results are in agreement with those of Lai et al. (2016), which showed that the starch properties depend on the coordinated expression of all genes within the pathway rather than on individual enzymes. Considering that amylose and amylopectin are synthesized through complex processes involving multiple starch-synthesizing enzymes (Zeeman et al., 2010; Lai et al., 2016), it is reasonable that the gene expression of one of these starch-synthesizing enzymes would not directly be associated with SR starch composition.

It was reported that the two types of SBE, SBEI and SBEII, differ in substrate specificity and expression patterns (Zeeman et al., 2010; Lai et al., 2016). However, the SBEI and SBEII detected in our RNA-seq showed similar gene expression patterns and levels (Figure 6). Thus, the functional differences and overlaps between the two types of SBE in sweet potato SRs merit future investigation.

### Interconversion of sucrose and starch may be a critical step in determining starch accumulation in SRs

Starch accumulation is a dynamic process, which includes the synthesis, degradation, transport, and conversion of sucrose and starch (Zeeman et al., 2010; Schreiber et al., 2014). Starch is synthesized in plant storage organs using the cleaved products of sucrose, which is the main photoassimilate from photosynthetic organs (Li and Zhang, 2003). Phenotypic data showed that there was no detectable sucrose in the SRs of YS33 at 65, 80, and 95 DAP, and in the SRs of XS22 at 95 and 110 DAP (Figure 2). The high levels of starch observed at these time points may result from the high rates of sucrose to starch conversion in the dry matter of YS33 and XS22 SRs at these stages.

Genes that affect sucrose cleavage and metabolism may be central regulators of starch accumulation in storage organs. Two pathways mediate sucrose cleavage in the cytosol; in one, sucrose is converted to glucose and fructose by invertase, and in the other, sucrose is converted to UDP-glucose and fructose by SuSy (Li and Zhang, 2003; Wind et al., 2010), and the UDP-glucose is then converted to glucose-1-phosphate by UGPase, for use in subsequent reactions related to starch synthesis (Figure S2; Li and Zhang, 2003; Schreiber et al., 2014). SuSy was reported to have high enzymatic activity in sweet potato SRs, and the expression of genes encoding SuSy was strongest amongst all of the sucrose metabolism genes in developing SRs. The SuSy pathway was shown to be the predominant pathway underlying sucrose cleavage related to starch accumulation in sweet potato (Li and Zhang, 2003). In our analysis, 13 SuSy and one UGPase encoding DEGs were detected, and most of these unigenes were expressed at very high levels in the SRs during all developmental stages examined, indicating that these genes have essential roles in sweet potato SRs. Furthermore, most of these genes were expressed at higher levels in the SRs of XS22 than in those of YS33 and SQ52-7. Combined with our phenotypic data that showed that XS22 SRs accumulated proportionately more starch and higher levels of glucose in the dry matter than did SQ52-7 and YS33, the high level of SuSy and UGPase in the SRs and the correspondingly high rates of sucrose cleavage and interconversion of sucrose to starch may be critical for the high levels of starch accumulation that occur during SR development in sweet potato.

### *Ibβfruct2*, which encodes vacuolar invertase, might be an important regulator of SR starch content

We found that *Ibβfruct2*, which encodes a vacuolar acid invertase, was expressed at high levels in SQ52-7, which had a low starch content, and at low levels in YS33, which had a high starch content. This negative correlation between *Ibβfruct2* expression and SR starch content indicates that the vacuolar acid invertase encoded by this gene regulates the starch content of sweet potato SRs. While there is little previous report of a vacuolar acid invertase regulating the starch properties of sweet potato SRs, Tanaka et al. (2009) demonstrated that *SRF1*, which encodes a Dof zing finger transcription factor, regulates the SR starch content through negatively regulating *Ibβfruct2* expression, but not by directly regulating the starch biosynthetic pathway. Previous studies also demonstrated that invertase might influence the starch content of storage organs (Tang et al., 1999; Draffehn et al., 2010; Li et al., 2013). However, the role of invertase in starch content regulation is unclear.

Here, we propose a model in which IbβFRUCT2 and its inhibitor regulate the starch content of sweet potato SRs (Figure S14). Firstly, we have determined that the SR water content is a key factor affecting the SR starch content. The water content of plant cells is under osmotic regulation by the vacuole. In SR cells, the vacuole-localized acid invertase catalyzes the hydrolysis of sucrose (which is transported into the vacuole through SUT) into fructose and glucose. As one molecule of sucrose is converted into two molecules of soluble monosaccharide in this reaction, the concentration of molecules in the vacuole increases, and the osmotic potential of the vacuole is reduced. To maintain the osmotic potential and molar concentration in the vacuole, water is transported into the vacuole, and the water content of the cell increases. Secondly, to maintain the sucrose concentration of the vacuole and the amount of catalytic substrate of vacuolar invertase, more sucrose will be transported into the vacuole, and the amount of sucrose converted into starch granules in the plastid will be reduced. At the same time, the degradation of starch granules is accelerated to generate more sucrose. Thus, the higher the activity of vacuolar invertase in SR cells, the more sucrose will be hydrolyzed to glucose and fructose in the vacuole, the higher the water content of the cell will be, and the lower the accumulation of starch granules. As the water content increases and the quantity of starch granules is reduced, the total starch content of sweet potato SRs is reduced. This can explain the negative correlation between the expression of *Ibβfruct2* and the SR starch content. Moreover, INVinh might also regulate the starch content by inhibiting invertase activity, so the expression of the detected INVinh encoding genes, which is inversely correlated with that of *Ibβfruct2* in the SR, might also be positively correlated with SR starch content.

In this analysis, we revealed the variation of total dry matter, starch accumulation, and sugar content in SRs derived from three genotypes during SR development, identified DEGs involved in starch and sucrose metabolism in sweet potato SRs, analyzed the expression pattern of these genes and the correlation between gene expression and variation in starch properties. Some of these genes had previously been reported in sweet potato, whereas others are novel genes that had not been detected. Our results reveal critical candidate loci involved in the biosynthesis and metabolism of starch and sugar during SR development, and the data produced in our study represent a useful resource for researchers aiming to decipher the physiological and molecular mechanisms underlying the starch and sugar properties of sweet potato SRs.

## Author contributions

KZ wrote the paper, designed and led much of the experimental work, and carried out the transcriptome analysis. ZW carried out much of the experimental work, performed trait measurements and qRT-PCR detection. DT assisted with field manipulation and plant management. HL, KLuo, YL and XW contributed to SR collection, trait measurement, and sample preparation. CL and JW provided sweet potato varieties for the study and helped with plant management. KLu and JD performed data analysis.

### Conflict of interest statement

The authors declare that the research was conducted in the absence of any commercial or financial relationships that could be construed as a potential conflict of interest.
