# Supplementary material for: Comparative Transcriptome Analysis Reveals Critical Function of Sucrose Metabolism Related-Enzymes in Starch Accumulation in the Storage Root of Sweet Potato
Source: Front Plant Sci. 2017 Jun 22;8:914. doi: 10.3389/fpls.2017.00914 (PMC5480015; doi:10.3389/fpls.2017.00914)
Supplement: Supplementary file 1 [file Table1.DOCX]

**Table S1** Primers used in qRT-PCR detection.

| Genes/Protein | Unigenes | Forward Primer | Reverse primer | Annealing temperature (℃) |
| --- | --- | --- | --- | --- |
| *IbAGPb1A* | comp83084_c0_seq1 | 5’- CAAGTAGATACTACTCTCTTGGGATTGCCA -3’ | 5’- CTTGAACATTGTGCTCCATCACAGCAGCAG -3’ | 55 |
| *IbAGPb1B* | comp37688_c0_seq1  comp46252_c0_seq1  comp53387_c0_seq1  comp68032_c0_seq1  comp67937_c0_seq1 | 5’- CAAGTAGATACTACTCGCCTGGGATTGTCT -3’ | 5’- CTTGAACATTGTGCTCTGTGATAACTGCGG -3’ | 55 |
| *IbAGPb2* | comp88605_c0_seq1 | 5’- CAAGTTGACACCTCTCTGCTAGGGCTTTCT -3’ | 5’- CTTGGACATTGTGGTCCTTCACTGCAGCGG -3’ | 55 |
| *IbAGPb3* | comp100284_c0_seq1 | 5’- GCTGTAGATACTTCGGTTTTGGGTCTTTCC -3’ | 5’- CCTGTATATAGAACTCCCTAGCTGAGGCAG -3’ | 55 |
| *IbAGPa1* | comp78401_c0_seq1 | 5’- AGCGTCTTGGGAATTATCCTCGGAGGTGGT -3’ | 5’- CTGGAACCAATTGGGGTTCTCAGGACTTTG -3’ | 55 |
| *IbAGPa2* | comp66680_c0_seq1 | 5’- CGGCAGCAATCGGAGCTCCGAAGCTCGC -3’ | 5’- GCTTTAGGGGAAACGATAATCGGAGCATTA -3’ | 55 |
| *IbGBSS* | comp84815_c0_seq1 | 5’- TCAAGCTGTGACTCACAATGGGTTGA -3’ | 5’- CAACAACCACACAGGTATCCCAAGC -3’ | 55 |
| *IbGBSS* | comp82416_c3_seq5 | 5'-TGGCATGTTCCGTGCGGTGG-3' | 5'-CCCGCGACCCTGGTGAGCTA-3' | 64 |
| *IbSSS* | comp87190_c0_seq4 | 5'-ACAGAGCCGGAGCCATCCCT-3 | '5'-GTGGGCGAGGCAAGGAAGCA-3' | 64 |
| *IbSSS* | comp89307_c0_seq2 | 5'-TGGAAACCCCGTCATTGCCCC-3' | 5'-GGCCCGTTTGCCTTCGACCA-3' | 60 |
| *IbSBEI* | comp82665_c1_seq1  comp82665_c0_seq2 | 5’- CAATGAGTGGGATGGGTCCAGTCAC -3’ | 5’- GTAGCATACTTTATCCAAGCTGGGA -3’ | 55 |
| *IbSBEII* | comp57909_c0_seq1  comp76071_c0_seq1 | 5’- ATCCGAACCCTCTTCTTTTAGAGTT-3’ | 5’- GACGAAATCCACATTGCTTTCACTG-3’ | 55 |
| ISA | comp81228_c0_seq2 | 5’- TCTGCTTGATCAGTCTCGCCGACTT-3’ | 5’- CCATTGGGGGCCAACAATCCTTCTC-3’ | 55 |
|  | comp89734_c1_seq4 | 5'-AGCCGGTGAATCAAGTGGCCTG -3' | 5'- ATTAACGGGTCCCCCACCAGCA-3' | 64 |
|  | comp88968_c0_seq1 | 5'-ACCAGCTCACCGACTCTGCCAT -3' | 5'-TGGCAGCAACAGCCGCAGATAC -3' | 64 |
| *IbSP* | comp79284_c0_seq2 | 5’- GATAATCAGAATGGATGTTTCATACC -3’ | 5’- CAGCATTTGCTTGGGCCTCACACGC -3’ | 55 |
|  | comp73377_c0_seq1 | 5'-AGCATTTGCCACATACATCCAGGC-3' | 5'-TTCCACTGGCCTCCATTCCAGCA-3' | 64 |
| AMY | comp26344_c0_seq1 | 5'-GGCGCCGAGGCGTGGTAACATTT-3' | 5'-ACACTGCATAATCATTCTTCTCCCA-3' | 55 |
| BMY | comp48829_c0_seq1 | 5'-CCGCCGCATTGGTGGAAGGA-3' | 5'-GCGCCGGAGTGGAGGGGATA-3' | 64 |
|  | comp69454_c1_seq3 | 5'-TCCCAAGCTCAAGATGTCCGGCT-3' | 5'-TCATACCACGGGAATGGCCTGCT-3' | 60 |
|  | comp70843_c0_seq1 | 5'-CTAGGCAGGGCAGGGCATGG-3' | 5'-CGGCTGAAAGTGCAGCAGCC-3' | 60 |
|  | comp87301_c0_seq1 | 5'-CAGTCTCCGGGGAGGGACCG-3' | 5'-GGCCGCAAACCTCCCCTCAC-3' | 64 |
|  | comp63470_c0_seq1 | 5'-CGGCGGCGGTGTCGATTTCA-3' | 5'-TCTGCTGCGGCTCCCCATCT-3' | 64 |
| DPE | comp77498_c0_seq6 | 5'-TTCCACCGCCAAGAAAGCCTGTG-3' | 5'-GTCCAGCTTCACTTGGCATGCGT-3' | 66 |
|  | comp79218_c0_seq1 | 5'-TGATGCGGGAATTTCCTTGGCGG-3' | 5'-TCCCACCACATCCCATGCACTGA-3' | 64 |
|  | comp87759_c1_seq1 | 5'-TCTTGCCACGACTGCTCCACTCT-3' | 5'-TCTCCTCCATTGCAGGGCGGATT-3' | 62.5 |
|  | comp85980_c0_seq2 | 5'-TGCCTTCAGCGAAACTGGTCAGC-3' | 5'-TTTGCTTCAGAGGGGACAGCCCA-3' | 62.5 |
| *IbSPS* | comp27340_c0_seq1 | 5'-GCACATGGATCAAGGTGGTGGCT-3' | 5'-TTTTCGCCCTTGTTCCCGTTCCC-3' | 64 |
|  | comp72263_c0_seq1 | 5'-GCCTTCCCGAAACACCACAAGCA-3' | 5'-GCAAACCGTTGTTCAGTGCCCGA-3' | 60 |
|  | comp79328_c0_seq4 | 5'-TGACATTCGGCAAAGGCTACGCA-3' | 5'-AGGCCCACGAGCAGGTCTTCATA-3' | 62.5 |
|  | comp86708_c0_seq2 | 5'-AGTGCGTTGGGCAGCTTCCATTT-3' | 5'-AAACGACATGGCAACGGAGAGCC-3' | 58 |
| *IbSuSy* | comp20955_c0_seq1 | 5'-ACCGCTGAGCGTGTCTTGGAGAT-3' | 5'-AACCTGGCCACCAGTGTCGGGATA-3' | 60 |
|  | comp47245_c0_seq1 | 5'-TCCTCACACGGTTCATCTGGGAA-3' | 5'-ACGGGGCTTGTCGAATGGTATGC-3' | 66 |
|  | comp63034_c0_seq1 | 5'-GCATCATGAGCTGCTGGCTGAGT-3' | 5'-ATTCCCAGACACCCGGCCTCAAA-3' | 62.5 |
|  | comp87700_c0_seq2 | 5'-ACGAACATATTCCCAGACCCCCG-3' | 5'-TGAAGCCTCACCAGCTCTTGGCT-3' | 62.5 |
|  | comp87700_c1_seq4 | 5'-ACGAAGGGAGCATTCGTGCAACC-3' | 5'-TGCCTGCTCGCCATGGTATGGAT-3' | 55 |
|  | comp37818_c0_seq1 | 5'-CCATGACAAGGAGAGCATGACACCC-3' | 5'-ACTCCTCCGCCTTTCTTAGCACG-3' | 60 |
|  | comp29963_c0_seq1 | 5'-TGGTACACTGTGTCACACCGAGCTT-3' | 5'-CTGCTGAGCTACAGGCCAAACCA-3' | 60 |
|  | comp60083_c0_seq1 | 5'-TGCCGCAATCTCGCAAGCTAAAGT-3' | 5'-AGCTCGATCGCCTTGAGATCCGT-3' | 64 |
|  | comp87700_c2_seq1 | 5’- GCCGTTCCATTGGCCGTCGAGTAAA -3’ | 5’- ACTCGAGGGTGACCAACAAAGACCA -3’ | 55 |
|  | comp65588_c0_seq1 | 5'-TCAGGTCCGGTTTTCCTTGCAGC-3' | 5'-TGCAACCAGCGGTTGGAGAGAGT-3' | 64 |
|  | comp69403_c0_seq1 | 5'-TGACGCGCACAAACTCCCAAACA-3' | 5'-ATTTGTGGCACAGGGGAAGGGGA-3' | 60 |
|  | comp71879_c0_seq1 | 5'-TTCCTTCCACGCCCAAGTTCCCT-3' | 5'-GATGCTTTGCCAGGTTCACCGGA-3' | 60 |
|  | comp78698_c0_seq1 | 5'-ATCATCCCGACGAGGCTGCTGAA-3' | 5'-TCTCTAGGTAGCGCCTAGTCTCGC-3' | 64 |
| UGPase | comp83799_c0_seq1 | 5’- GAATCCTTCTATTGAATTAGGACCTG-3’ | 5’- TCTGGAATTTCTAACTTGCCAGATT-3’ | 55 |
| GS | comp72115_c0_seq3 | 5'-CGGGTGGCCTTGCAGACACA-3' | 5'-TCTGCACGGTAGCGTCCCAT-3' | 60 |
|  | comp66443_c0_seq2 | 5'-GCTGCAGGTACCGCTCAAGGC-3' | 5'-AAGTCGCAGCACTCCGCGTC-3' | 60 |
| *IbSPP* | comp81691_c1_seq1 | 5'-TGACGATGAGGTGCGTGAGCAGA-3' | 5'-ATTCCGGCAGGGTGATGGGTAGT-3' | 58 |
| *IbPGI* | comp85765_c0_seq1 | 5'-ACTTCTTTGCACAGCCAGACGCC-3' | 5'-AAACCCTTGCACAGCAACCCTGT-3' | 58 |
|  | comp88695_c0_seq1 | 5'-AAGCCGCAGGAGAAGTTCTAGCCC-3' | 5'-CGTTGGCTGCCATGTGCGCGATTA-3' | 55 |
| UGDH | comp87686_c0_seq4 | 5'-ACGAGGCAACCAAAGATGCCCAC-3' | 5'-CTTGAGCCAAGCATCCAACGGCT-3' | 55 |
| GAE | comp84725_c1_seq1 | 5'-CACCAAGAAAGCCGGCGAGGAAA-3' | 5'-GACGCCACGCACCCTTTAACGAT-3' | 55 |
| *Ibβfruct2* | comp85641_c0_seq1 | 5’- CCATTCCGCCGGAGTTGAGATCCGTGA -3’ | 5’- CTCCCCGGAACGCCTTCTCGGAAAC -3’ | 55 |
| INVinh | comp59423_c0_seq1 | 5'-AAAGTCGAAGCCGGTGGAGTGGA -3' | 5'-TCTGCAGCTTAAACGCAGCCTCG-3' | 60 |
|  | comp70068_c0_seq1 | 5'-AGCCCCTCAGCCAGTGCTACTTT-3' | 5'-TGACGTGGCCACACCAGAGAGTT-3' | 60 |
|  | comp80373_c0_seq2 | 5'-CGTGATGGAAACCGCGTCGACTT-3' | 5'-TCAAGATGACTCAGCTCGCCGGA-3' | 60 |
|  | comp67966_c0_seq3 | 5'-ACGTGGGGGAGGCGTTGAGGTATT-3' | 5'-TCAAACGTGGCGTAGTTCCTGGCCG-3' | 55 |
| SUT | comp37930_c0_seq1 | 5'-ATGGCCAAGGAGGTCTACGGCG -3' | 5'-ACCGTCATGCCCAAACAAATCGC -3' | 60 |
|  | comp78671_c0_seq1 | 5'-AATGAAAACCACCAGCAGGTACTGTAG -3' | 5'-ACTGTTTGGCGGCGGCAATTT -3' | 60 |
|  | comp81616_c0_seq5 | 5'-GTTTTCGTCGCCGGCTTCTGGAT-3' | 5'-GTCGCAGGCTTTCGTCTTCGTGA-3' | 60 |
|  | comp62788_c7_seq1 | 5'-AGGGCGACGGAGATGAAGAAGCA-3' | 5'-AATGCGAGCGTCAAACGCACTCT-3' | 60 |
| G6PPT | comp83665_c1_seq1 | 5'-TCTTCCGGACACCAGTCCAGCCTGT-3' | 5'-GCAGCTCGCAGCTTGCTTCATCACC-3' | 55 |
| SWEET | comp82605_c0_seq1 | 5'-TGGTGCACCCTGTGGATTCAGGGA-3' | 5'-TCACTTGATGGACTCAGCCGGCCA-3' | 55 |
