# Supplementary material for: Comparative Transcriptome Analysis Reveals Critical Function of Sucrose Metabolism Related-Enzymes in Starch Accumulation in the Storage Root of Sweet Potato
Source: Front Plant Sci. 2017 Jun 22;8:914. doi: 10.3389/fpls.2017.00914 (PMC5480015; doi:10.3389/fpls.2017.00914)
Supplement: Supplementary file 2 [file Table2.DOCX]

**Table S2** Dry matter and starch content as a percentage of the total fresh weight in the SRs of the three genotypes examined at the harvest stage in 2011–2013.

| Accessions | Dry matter (%) | | | | Starch content (%) | | | | Scope of starch content  (%) |
| --- | --- | --- | --- | --- | --- | --- | --- | --- | --- |
|  | 2011 | 2012 | 2013 | Mean value | 2011 | 2012 | 2013 | Mean value |  |
| YS33 | 35.471±0.050 | 36.756±0.459 | 31.179±0.034 | 34.469±2.385 | 24.495±0.043 | 25.611±0.399 | 20.763±0.029 | 23.623±2.073 | 20–25 |
| XS22 | 28.493±0.290 | 26.376±0.086 | 29.001±0.352 | 27.957±1.137 | 18.427±0.252 | 16.586±0.075 | 18.869±0.306 | 17.961±0.989 | 15–20 |
| SQ52-7 | 12.941±0.061 | 12.446±0.174 | 15.788±1.273 | 13.725±1.473 | 4.906±0.053 | 4.476±0.151 | 7.381±1.107 | 5.588±1.280 | <10 |

2011, 2012, and 2013: the year in which the traits were measured. Error bars indicate the standard deviation from mean of values obtained in the three years.
