# Supplementary material for: Comparative Transcriptome Analysis Reveals Critical Function of Sucrose Metabolism Related-Enzymes in Starch Accumulation in the Storage Root of Sweet Potato
Source: Front Plant Sci. 2017 Jun 22;8:914. doi: 10.3389/fpls.2017.00914 (PMC5480015; doi:10.3389/fpls.2017.00914)
Supplement: Supplementary file 5 [file Table5.DOCX]

**Table S5** Sequence length distributions of contig sequences in the sweet potato SR transcriptome.

|  | All  (>=200 bp) | >=500  bp | >=1000  bp | N50 | N90 | Total  Length | Max  Length | Min  Length | Average  Length |
| --- | --- | --- | --- | --- | --- | --- | --- | --- | --- |
| Transcript | 241386 | 132872 | 79631 | 1640 | 388 | 235082287 | 24052 | 201 | 973.89 |
| Unigene | 112336 | 39489 | 18979 | 1061 | 267 | 74410563 | 24052 | 201 | 662.39 |
