# Supplementary material for: Comparative Transcriptome Analysis Reveals Critical Function of Sucrose Metabolism Related-Enzymes in Starch Accumulation in the Storage Root of Sweet Potato
Source: Front Plant Sci. 2017 Jun 22;8:914. doi: 10.3389/fpls.2017.00914 (PMC5480015; doi:10.3389/fpls.2017.00914)
Supplement: Supplementary file 6 [file Table6.DOCX]

**Table S6** Overview of unigene annotation statistics derived from the Nr, Swiss-Prot, TrEMBL, CDD, Pfam, and KOG databases.

|  | Total | Nr | Swiss-Prot | TrEMBL | CDD | pfam | KOG |
| --- | --- | --- | --- | --- | --- | --- | --- |
| Unigene number | 112336 | 35335 | 21223 | 35096 | 20933 | 32907 | 10711 |
| Percentage | 100% | 31.45% | 18.89% | 31.24% | 18.63% | 29.29% | 9.53% |
