# Supplementary material for: Comparative Transcriptome Analysis Reveals Critical Function of Sucrose Metabolism Related-Enzymes in Starch Accumulation in the Storage Root of Sweet Potato
Source: Front Plant Sci. 2017 Jun 22;8:914. doi: 10.3389/fpls.2017.00914 (PMC5480015; doi:10.3389/fpls.2017.00914)
Supplement: Supplementary file 7 [file Table7.DOCX]

**Table S7** Gene expression abundance in the SRs of the three genotypes examined at different developmental stages.

| Samples | Number of expression transcript | Min. | 1st Qu. | Median | Mean | 3rd Qu. | Max. | Sd. | Sum. |
| --- | --- | --- | --- | --- | --- | --- | --- | --- | --- |
| SQ52-7 65DAP | 67224 | 0 | 0.16 | 0.42 | 8.69 | 2.84 | 58234.03 | 275.38 | 584348.67 |
| SQ52-7 80DAP | 68626 | 0 | 0.14 | 0.35 | 8.14 | 2.18 | 71040.68 | 358.95 | 558768.35 |
| SQ52-7 95DAP | 67990 | 0 | 0.17 | 0.45 | 8.19 | 2.46 | 56015.73 | 274.93 | 556667.67 |
| SQ52-7 110DAP | 77498 | 0 | 0.21 | 0.54 | 7.17 | 2.38 | 35906.04 | 194.6 | 555303.25 |
| SQ52-7 125DAP | 66539 | 0 | 0.16 | 0.41 | 8.2 | 2.36 | 73251.71 | 348.24 | 545962.46 |
| XS22 65DAP | 74509 | 0 | 0.16 | 0.4 | 7.65 | 2.52 | 55775.11 | 275.78 | 570164.45 |
| XS22 80DAP | 78697 | 0 | 0.18 | 0.45 | 7.38 | 2.61 | 51424.66 | 240.39 | 581137.8 |
| XS22 95DAP | 60691 | 0 | 0.15 | 0.41 | 7.85 | 2.07 | 116297.03 | 524.17 | 476632.2 |
| XS22 110DAP | 71643 | 0.01 | 0.16 | 0.39 | 7.55 | 2.37 | 72417.22 | 354.16 | 540700.7 |
| XS22 125DAP | 80261 | 0 | 0.19 | 0.44 | 6.96 | 2.43 | 57157.28 | 244.64 | 558527.39 |
| YS33 65DAP | 67712 | 0 | 0.17 | 0.45 | 9.08 | 2.63 | 59801.08 | 309.8 | 614862.37 |
| YS33 80DAP | 49916 | 0 | 0.1 | 0.26 | 7.64 | 1.4 | 123745.39 | 732.45 | 381215.09 |
| YS33 95DAP | 80330 | 0.01 | 0.19 | 0.47 | 6.79 | 2.04 | 51405.42 | 264.64 | 545689.07 |
| YS33 110DAP | 60505 | 0 | 0.15 | 0.4 | 8.28 | 2.06 | 99270.57 | 512.18 | 501077.5 |
| YS33 125DAP | 70672 | 0.01 | 0.16 | 0.41 | 8.11 | 2.69 | 61904.7 | 290.88 | 573468.65 |
