# Supplementary material for: Comparative Transcriptome Analysis Reveals Critical Function of Sucrose Metabolism Related-Enzymes in Starch Accumulation in the Storage Root of Sweet Potato
Source: Front Plant Sci. 2017 Jun 22;8:914. doi: 10.3389/fpls.2017.00914 (PMC5480015; doi:10.3389/fpls.2017.00914)
Supplement: Supplementary file 8 [file Table8.DOC]

**Table S8** DEGs in the SR transcriptome involved in starch and sucrose metabolism.

| Enzyme/Protein | EC Number | Isoforms/ Gene abbreviation | Unigenes | Accession number | Function | Reaction | |
| --- | --- | --- | --- | --- | --- | --- | --- |
| DEGs involved in starch granule formation and degradation | | | | | | | |
| ADP-glucose pyrophosphorylase (AGPase)  Glucose-1-phosphate adenylyltransferase | EC 2.7.7.27 | Large (β) subunit 1/*IbAGPb1A* | comp83084_c0_seq1 | AF068260.1 | Synthesis of ADP-glucose, the substrate for starch synthesis⇄ | | α-D-glucose-1-phosphate +ATP⇄ADP-glucose+diphosphate (PPi) |
|  |  | Large (β) subunit 2/*IbAGPb1B* | comp37688_c0_seq1  comp46252_c0_seq1  comp53387_c0_seq1  comp68032_c0_seq1  comp67937_c0_seq1 | JQ797693.1 |  |  |  |
|  |  | Large (β) subunit 3/*IbAGPb2* | comp88605_c0_seq1 | JQ797694.1 |  |  |  |
|  |  | Large (β) subunit 4 /*IbAGPb3* | comp100284_c0_seq1  comp74242_c0_seq1 | AB271016.1 |  |  |  |
|  |  | Small (α) subunit 1/*IbAGPa1* | comp78401_c0_seq1 | Z79635.1 |  |  |  |
|  |  | Small (α) subunit 2/*IbAGPa2* | comp66680_c0_seq1 | Z79636.1 |  |  |  |
| Granule-bound starch synthase (GBSS) | EC 2.4.1.242 | GBSSI/*IbGBSS* | comp84815_c0_seq1 | AB071604.1 | Amylose biosynthesis | | NDP-glucose + (1,4-α-D-glucosyl)_n_ =NDP + (1,4-α-D-glucosyl)_n+1_ |
|  |  |  | comp82416_c3_seq5 | AF068834.1* |  |  |  |
| Soluble starch synthase (SSS) | EC 2.4.1.21 | SSI/*IbSSS* | comp87190_c0_seq4 |  | Elongation of a-1, 4-linked glucan chain | | ADP-α-D-glucose + [(1→4)-α-D-glucosyl]_n_= ADP + [(1→4)-α-D-glucosyl]_n+1_ |
|  |  | SSIII/*IbSSS* | comp89307_c0_seq2 |  |  |  |  |
| Starch-branching enzyme (SBE) (1,4-alpha-glucan-branching enzyme)  amylo-(1,4→1,6)-transglycosylase; Q-enzyme | EC 2.4.1.18 | SBEI/*IbSBEI* | comp82665_c1_seq1  comp82665_c0_seq2 | AB194723.1 | Branching through a-1, 6 linkage | | Transfers a segment of a (1→4)-α-D-glucan chain to a primary hydroxy group in a similar glucan chain |
|  |  | SBEII/*IbSBEII* | comp57909_c0_seq1  comp76071_c0_seq1 | AB071286.1 |  |  |  |
| Isoamylase (ISA)  (Glycogen α-1,6-glucanohydrolase) | EC 3.2.1.68 | DBE/ISA, *IbIsal* | comp81228_c0_seq2 | DQ074643.1 | Implicated in amylopectin synthesis or starch degradation | | Hydrolysis of (1→6)-α-D-glucosidic branch linkages in glycogen, amylopectin and their β-limit dextrins |
|  |  |  | comp89734_c1_seq4 |  |  |  |  |
|  |  |  | comp88968_c0_seq1 |  |  |  |  |
| Starch phosphorylase (Pho)  (α-1,4 -glucan phosphorylase) | EC 2.4.1.1 | SP/*IbSP* | comp79284_c0_seq2  comp73377_c0_seq1 | M64362.1 | Extending glucan chains using glucose-1-phosphate as a substrate (synthesis) or releasing glucose-1-phosphate from glycogen or starch (degradation) | | [(1->4)-α-D-glucosyl]_n_+phosphate ⇄ [(1->4)- α-D-glucosyl]_n-1_ +α-D-glucose 1-phosphate |
| α-amylase (AMY) | EC 3.2.1.1 | AMY | comp26344_c0_seq1 |  | Starch degrading | | Endohydrolysis of (1→4)-α-D-glucosidic linkages in polysaccharides containing three or more (1→4)-α-linked D-glucose units |
| β-amylase (BMY)  （α-1,4-D-glucan maltohydrolase） | EC 3.2.1.2 | BMY | comp48829_c0_seq1 |  | Hydrolysis of the linear chains in starch degradation | | Hydrolysis of (1→4)-α-D-glucosidic linkages in polysaccharides so as to remove successive maltose units from the non-reducing ends of the chains |
|  |  |  | comp69454_c1_seq3 | D01022.1 |  |  |  |
|  |  |  | comp70843_c0_seq1 |  |  |  |  |
|  |  |  | comp87301_c0_seq1 |  |  |  |  |
|  |  |  | comp63470_c0_seq1 |  |  |  |  |
| 4-α-glucanotransferase  (Disproportionating enzyme (DPE), D-enzyme) | EC 2.4.1.25 |  | comp77498_c0_seq6 |  | Cleavage and transfer of a-1, 4- linked glucan | | Transfers a segment of a (1→4)-α-D-glucan to a new position in an acceptor, which may be glucose or a (1→4)-α-D-glucan |
|  |  |  | comp79218_c0_seq1 |  |  |  |  |
|  |  | DPE1 | comp87759_c1_seq1 | KF020877.1 |  |  |  |
|  |  |  | comp85980_c0_seq2 |  |  |  |  |
| DEGs involved in sucrose synthesis and conversion | | | | | | | |
| sucrose-phosphate synthase | EC 2.4.1.14 | SPS/*IbSPS* | comp27340_c0_seq1 |  | Catalyzing reversible step acts as the key regulatory control point in sucrose biosynthesis | | UDP-glucose +D-fructose 6-phosphate ⇄ UDP + sucrose 6-phosphate |
|  |  |  | comp72263_c0_seq1 | AF135800.1* |  |  |  |
|  |  |  | comp79328_c0_seq4 |  |  |  |  |
|  |  |  | comp86708_c0_seq2 | AF439861.1 |  |  |  |
| Sucrose synthase | EC 2.4.1.13 | SuSy/*IbSuSy* | comp20955_c0_seq1 |  | Providing UDP-glucose for starch synthesis | | NDP-glucose +D-fructose ⇄ NDP + sucrose |
|  |  |  | comp47245_c0_seq1 | EU908020.1* |  |  |  |
|  |  |  | comp63034_c0_seq1 |  |  |  |  |
|  |  |  | comp87700_c0_seq2 |  |  |  |  |
|  |  |  | comp87700_c1_seq4 | EU908020.1 |  |  |  |
|  |  |  | comp37818_c0_seq1 |  |  |  |  |
|  |  |  | comp29963_c0_seq1 |  |  |  |  |
|  |  |  | comp60083_c0_seq1 | EU908020.1* |  |  |  |
|  |  |  | comp87700_c2_seq1 | EU908020.1* |  |  |  |
|  |  |  | comp65588_c0_seq1 |  |  |  |  |
|  |  |  | comp69403_c0_seq1 |  |  |  |  |
|  |  |  | comp71879_c0_seq1 |  |  |  |  |
|  |  |  | comp78698_c0_seq1 |  |  |  |  |
| UDP-glucose pyrophosphorylase  (UTP-glucose-1-phosphate uridylyltransferase) | EC 2.7.7.9 | UGPase | comp83799_c0_seq1 | EU863220.1 | Converting UDP-glucose  to glucose-1-phosphate for sucrose and starch synthesis | | Glucose-1-phosphate + UTP ⇄ UDP-glucose + pyrophosphate |
| DEGs involved in sucrose metabolism | | | | | | | |
| Glycogen (starch) synthase  （UDP-glucose-glycogen glucosyltransferase） | EC 2.4.1.11 | GS | comp72115_c0_seq3 |  | Converting glucose to glycogen | UDP-glucose+[(1→4)-α-D-glucosyl]_n_=UDP+[(1→4)-α-D-glucosyl]_n+1_ | |
|  |  |  | comp66443_c0_seq2 |  |  |  |  |
| Sucrose-phosphatase  (sucrose-phosphate phosphatase) | EC 3.1.3.24 | SPP/IbSPP | comp81691_c1_seq1 |  |  | sucrose 6-phosphate + H_2_O ⇄sucrose + phosphate | |
| glucose-6-phosphate isomerase | EC 5.3.1.9 | PGI | comp85765_c0_seq1  comp88695_c0_seq1 |  |  | glucose 6-phosphate ⇄ fructose 6-phosphate | |
| UDP-glucose 6-dehydrogenase (uridine diphosphate glucose dehydrogenase) | EC 1.1.1.22 | UGDH | comp87686_c0_seq4 | KC509923.1 |  | UDP-glucose + 2 NAD^+^+H_2_O = UDP-glucuronate+2 NADH | |
| UDP-glucuronate 4-epimerase | EC 5.1.3.6 | GAE | comp84725_c1_seq1 |  |  | UDP-glucuronate ⇄ UDP-D-galacturonate | |
| Soluble acid invertase  β-fructofuranosidase | EC 3.2.1.26 | IbβFRUCT2/*Ibβfruct2* | comp85641_c0_seq1 | AY037937.1 |  | Hydrolyze sucrose into  fructose and glucose | |
| Invertase inhibitor |  | INVinh | comp59423_c0_seq1  comp70068_c0_seq1  comp80373_c0_seq2  comp67966_c0_seq3 | AF529166.1* | Inhibition of invertase activity |  | |
| DEGs encoding transporters | | | | | | | |
| Sucrose transporter (SUT) （Sucrose transport protein） |  |  | comp37930_c0_seq1 |  | Transport sucrose from source to sink tissue | Sucrose transmembrane transporter | |
|  |  |  | comp78671_c0_seq1 |  |  |  |  |
|  |  | SUT2x  SUT2y | comp81616_c0_seq5 | GQ979979.1  GU395494.1 |  |  |  |
|  |  | SUT3 | comp62788_c7_seq1 | GQ979981.1* |  |  |  |
| Glucose-6-phosphate/phosphate translocator |  | G6PPT | comp83665_c1_seq1 |  | Transport of Glucose 6-phosphate into plastids of heterotrophic tissues where it can be used as a carbon source for starch biosynthesis | Glucose 6-phosphate transporter | |
| Bidirectional sugar transporter |  | SWEET | comp82605_c0_seq1 |  |  | Transport of sugar across the plasma membrane | |

* The unigenes showed partial sequence similar with the genes under the accession number.
