# Supplementary material for: Comparative Transcriptome Analysis Reveals Critical Function of Sucrose Metabolism Related-Enzymes in Starch Accumulation in the Storage Root of Sweet Potato
Source: Front Plant Sci. 2017 Jun 22;8:914. doi: 10.3389/fpls.2017.00914 (PMC5480015; doi:10.3389/fpls.2017.00914)
Supplement: Supplementary file 11 [file Image2.PDF]

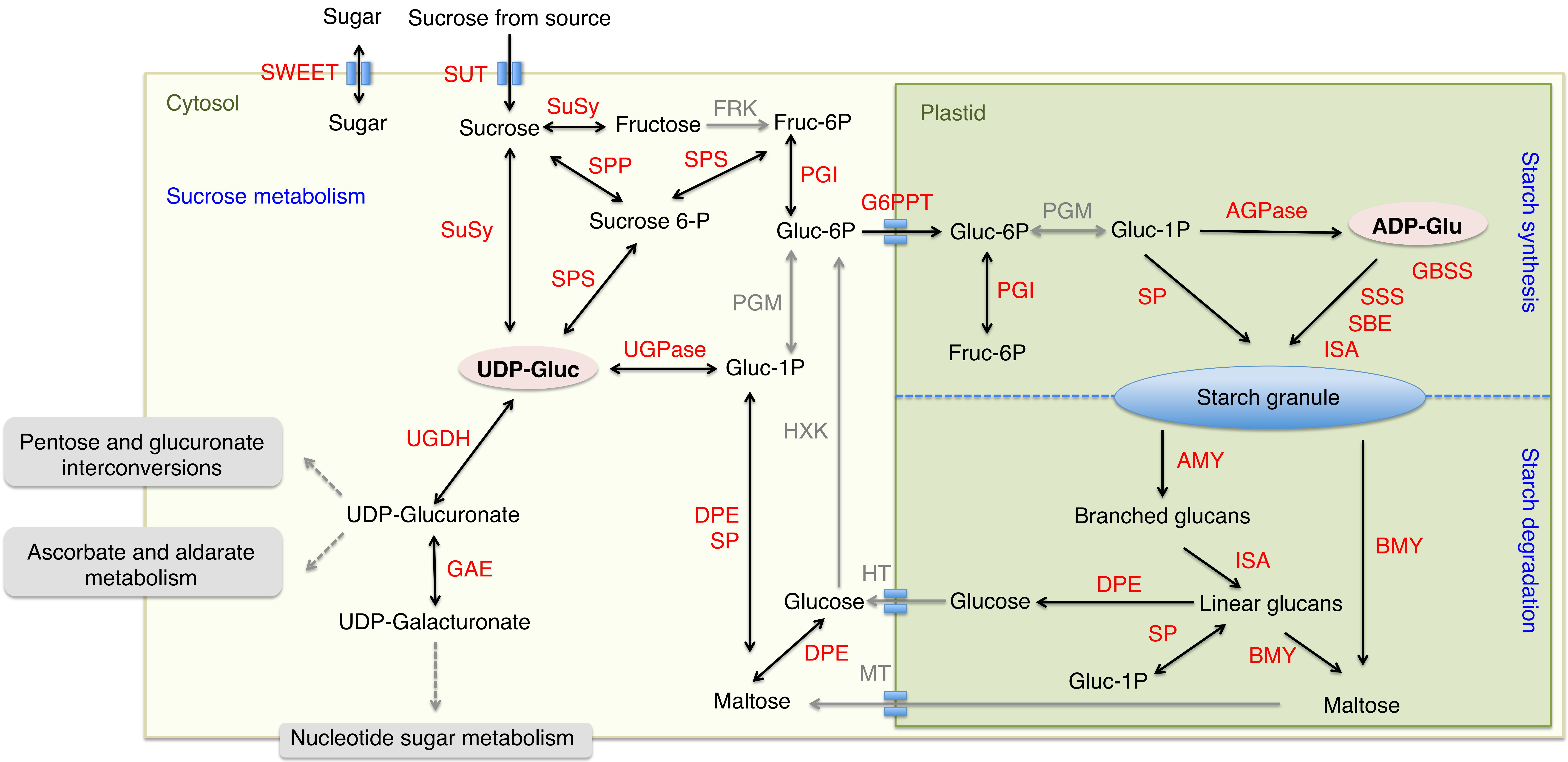

Figure S2 Illustration of the roles of enzymes and transporters involved in starch and sucrose metabolism in the SRs of sweet potato.

Modified from Ferreira and Sonnewald (2012) and Schreiber et al. (2014). The proteins encoded by DEGs detected in this study are in red font. AGPase, ADP-glucose pyrophosphorylase (EC 2.7.7.27); AMY, α-amylase (EC 3.2.1.1); BMY, β-amylase (EC 3.2.1.2); DPE, 4-α -glucanotransferase (EC 2.4.1.25); FRK, fructokinase (EC 2.7.1.4); G6PPT, glucose-6-phosphate/phosphate translocator; GAE, UDP-glucuronate 4-epimerase (EC 5.1.3.6); GBSS, granule bound starch synthase (EC 2.4.1.21); HT, hexose transporter; HXK, hexokinase (EC 2.7.1.1); ISA, isoamylase (EC 3.2.1.68); MT, maltose transporter; PGI, glucose-6-phosphate isomerase (EC 5.3.1.9); PGM, phosphoglucomutase (EC 5.4.2.2); SBE, starch branching enzyme (EC 2.4.1.18); SP, starch phosphorylase (EC 2.4.1.1); SPP, sucrose-phosphate phosphatase (EC 3.1.3.24); SPS, sucrose phosphate synthase (EC2.4.1.14); SSS, soluble starch synthase (EC 2.4.1.21); SuSy, sucrose synthase (EC 2.4.1.13); SUT, sucrose transporter; SWEET, bidirectional sugar transporter; UGDH, UDP-glucose 6-dehydrogenase (EC 1.1.1.22); UGPase, UDP-glucose pyrophosphorylase (EC 2.7.7.9); ADP-Gluc, ADP-glucose; Fruc-6P, fructose-6-phosphate; Gluc-1P, glucose-1-phosphate; Gluc-6P, glucose-6-phosphate; sucrose 6-P, sucrose-6- phosphate; UDP-Gluc, UDP-glucose.
