# Supplementary material for: Comparative Transcriptome Analysis Reveals Critical Function of Sucrose Metabolism Related-Enzymes in Starch Accumulation in the Storage Root of Sweet Potato
Source: Front Plant Sci. 2017 Jun 22;8:914. doi: 10.3389/fpls.2017.00914 (PMC5480015; doi:10.3389/fpls.2017.00914)
Supplement: Supplementary file 12 [file Image3.PDF]

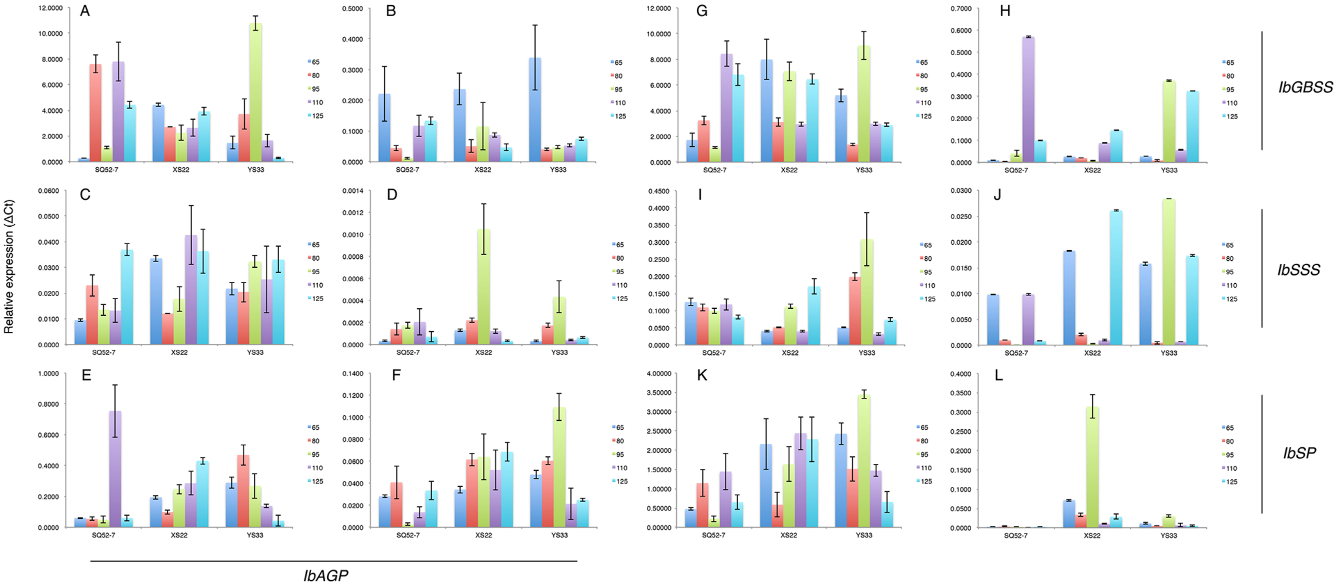

Figure S3 QRT-PCR analysis of the expression patterns of genes encoding enzymes involved in starch granule formation during SR development in the three sweet potato genotypes examined.

A–F, the expression patterns of IbAGPb1A, IbAGPb1B, IbAGPb2, IbAGPb3, IbAGPa1, and IbAGPa2 unigenes (as shown in Table S6); G–L, the expression pattern of the IbGBSS unigene comp84815_c0_seq1, IbGBSS unigene comp82416_c3_seq5, IbSSS unigene comp87190_c0_seq4, IbSSS unigene comp89307_c0_seq2, IbSP unigene comp79284_c0_seq2, and IbSP unigene comp73377_c0_seq1. The relative gene expression level was quantified using the ΔCt method. Error bars represent SEM of three independent biological replicates.
