# Supplementary material for: Comparative Transcriptome Analysis Reveals Critical Function of Sucrose Metabolism Related-Enzymes in Starch Accumulation in the Storage Root of Sweet Potato
Source: Front Plant Sci. 2017 Jun 22;8:914. doi: 10.3389/fpls.2017.00914 (PMC5480015; doi:10.3389/fpls.2017.00914)
Supplement: Supplementary file 13 [file Image4.PDF]

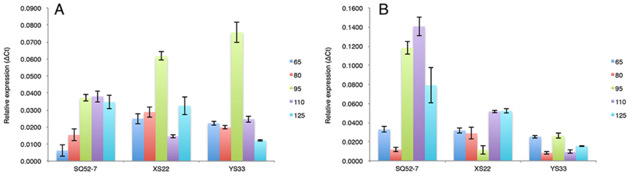

Figure S4 Expression pattern of GS encoding unigenes.

The expression pattern of comp66443_c0_seq2 (A) and comp72115_c0_seq3 (B), as detected by qRT-PCR.
