# Supplementary material for: Comparative Transcriptome Analysis Reveals Critical Function of Sucrose Metabolism Related-Enzymes in Starch Accumulation in the Storage Root of Sweet Potato
Source: Front Plant Sci. 2017 Jun 22;8:914. doi: 10.3389/fpls.2017.00914 (PMC5480015; doi:10.3389/fpls.2017.00914)
Supplement: Supplementary file 14 [file Image5.PDF]

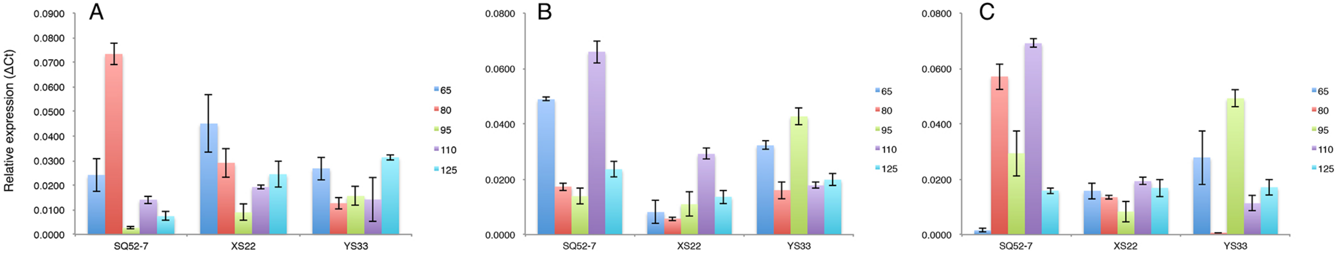

Figure S5 Expression patterns of the IbISA unigenes.

QRT-PCR analysis of the expression patterns of the IbISA unigenes comp81228_c0_seq2 (A), comp89734_c1_seq4 (B), and comp88968_c0_seq1 (C).
