# Supplementary material for: Comparative Transcriptome Analysis Reveals Critical Function of Sucrose Metabolism Related-Enzymes in Starch Accumulation in the Storage Root of Sweet Potato
Source: Front Plant Sci. 2017 Jun 22;8:914. doi: 10.3389/fpls.2017.00914 (PMC5480015; doi:10.3389/fpls.2017.00914)
Supplement: Supplementary file 15 [file Image6.PDF]

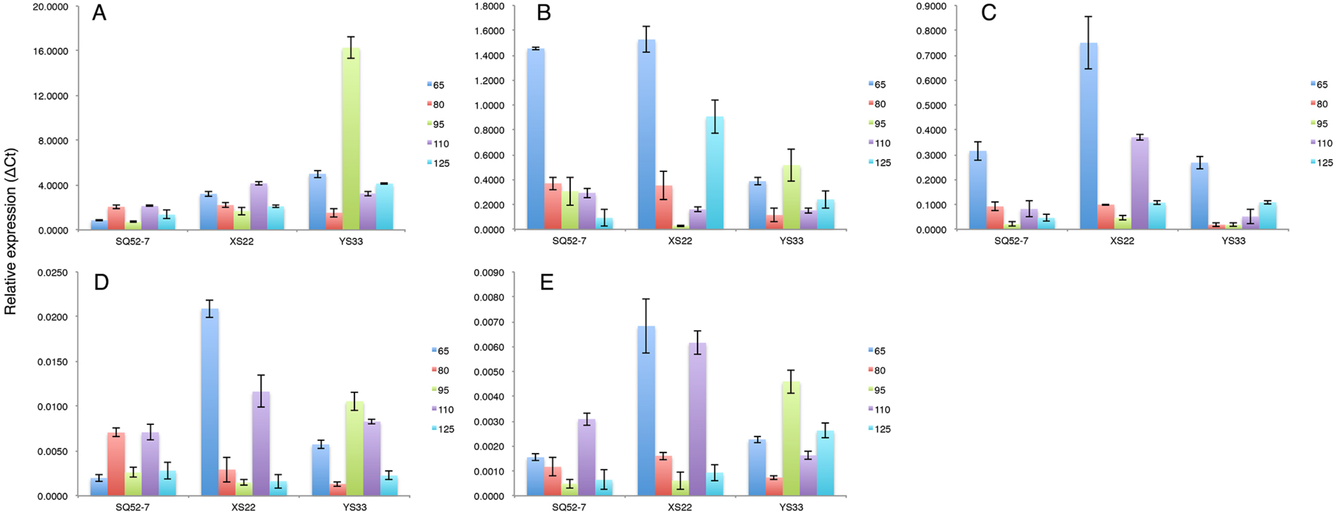


Figure S6 Expression patterns of β-amylase encoding unigenes.

The expression patterns of unigenes comp69454_c1_seq3 (A), comp70843_c0_seq1 (B), comp87301_c0_seq1 (C), comp63470_c0_seq1 (D), and comp48829_c0_seq1 (E), as detected by qRT-PCR.
