# Supplementary material for: Comparative Transcriptome Analysis Reveals Critical Function of Sucrose Metabolism Related-Enzymes in Starch Accumulation in the Storage Root of Sweet Potato
Source: Front Plant Sci. 2017 Jun 22;8:914. doi: 10.3389/fpls.2017.00914 (PMC5480015; doi:10.3389/fpls.2017.00914)
Supplement: Supplementary file 16 [file Image7.PDF]

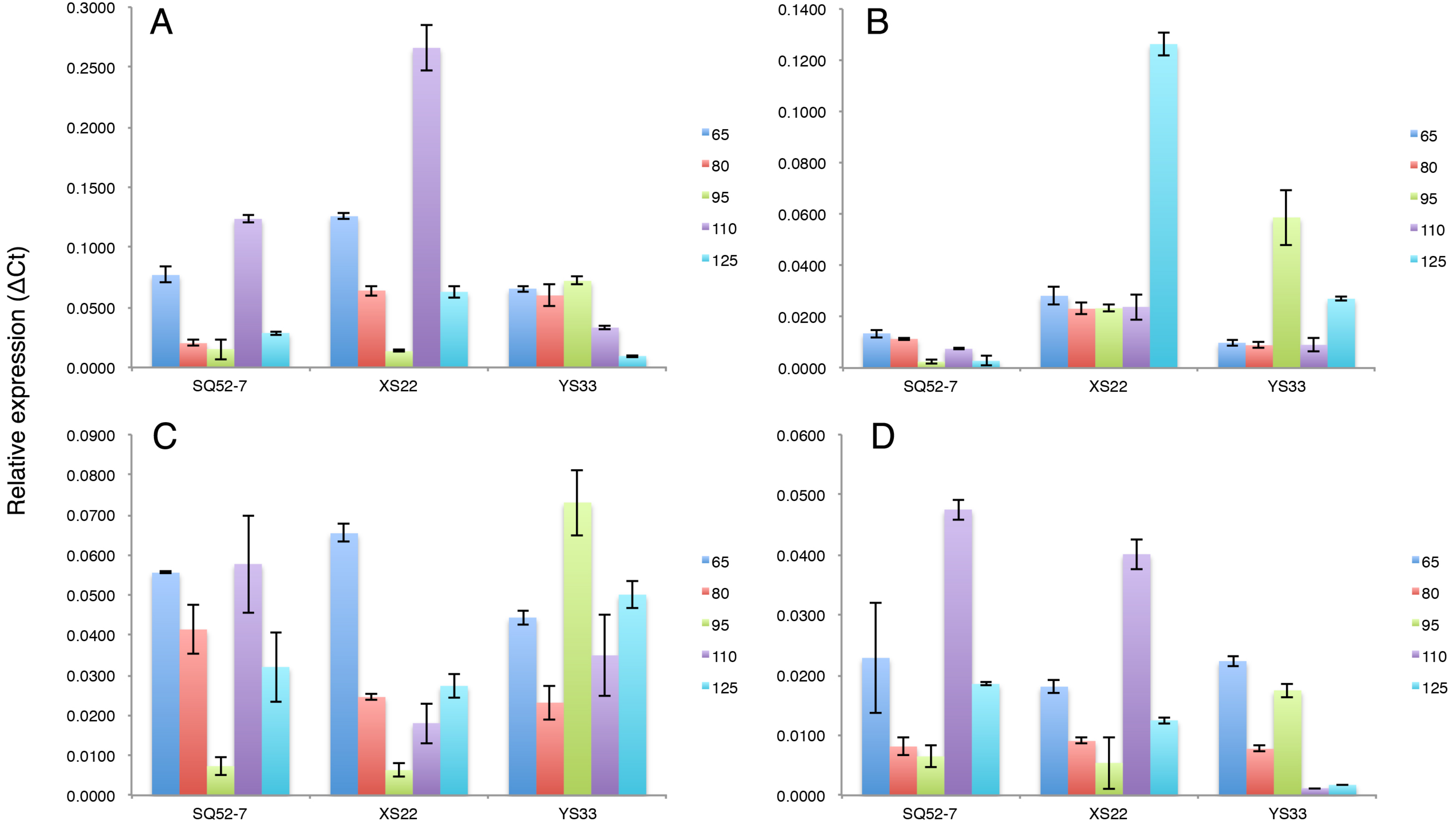

Figure S7 Expression patterns of DPE encoding unigenes.

QRT-PCR analysis of the unigenes comp77498_c0_seq6 (A), comp87759_c1_seq1 (B), comp85980_c0_seq2 (C), and comp79218_c0_seq1 (D).
