# Supplementary material for: Comparative Transcriptome Analysis Reveals Critical Function of Sucrose Metabolism Related-Enzymes in Starch Accumulation in the Storage Root of Sweet Potato
Source: Front Plant Sci. 2017 Jun 22;8:914. doi: 10.3389/fpls.2017.00914 (PMC5480015; doi:10.3389/fpls.2017.00914)
Supplement: Supplementary file 17 [file Image8.PDF]

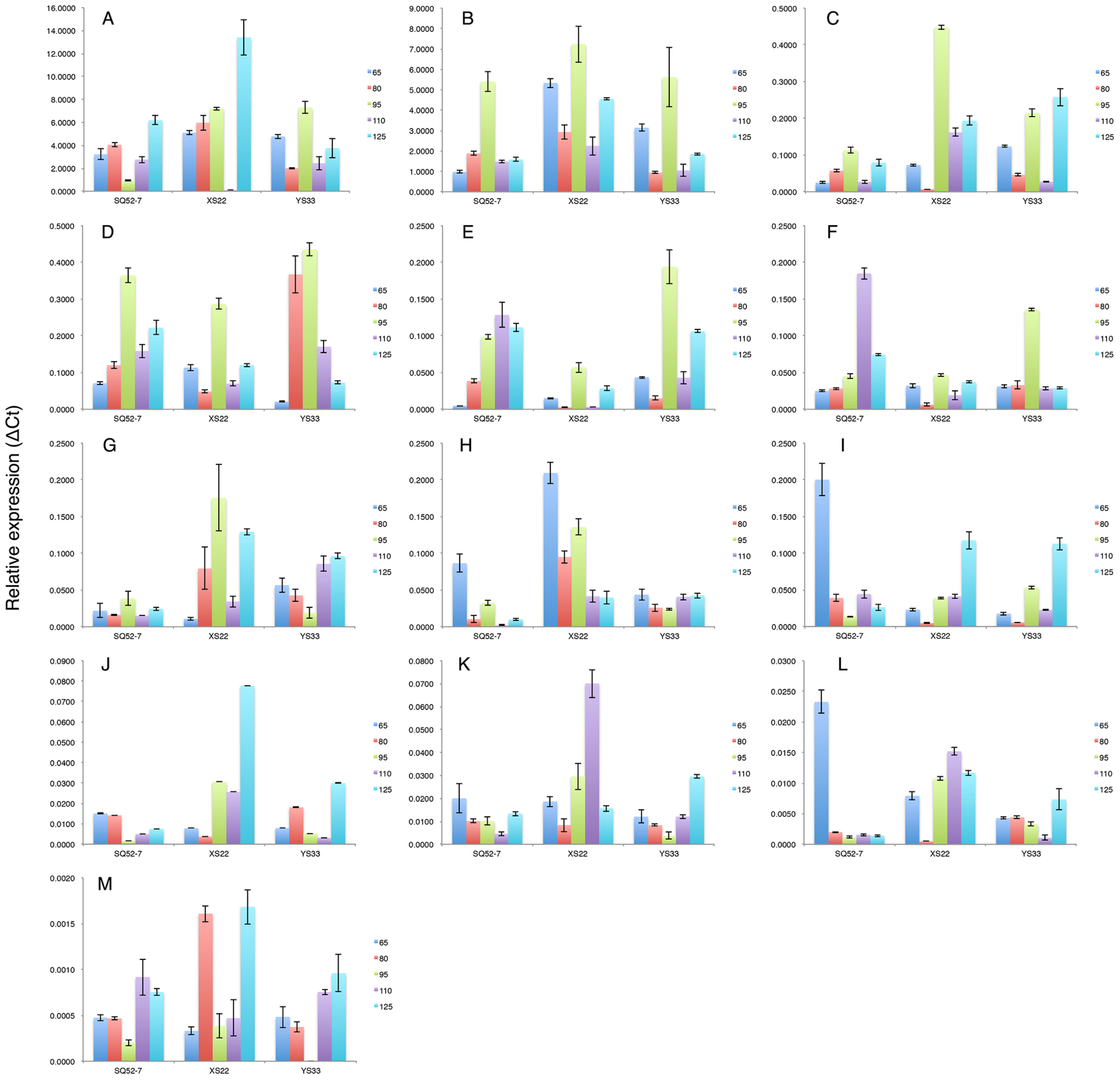

Figure S8 Expression pattern of SuSy encoding unigenes, as detected by qRT-PCR.

A, the expression pattern of comp87700_c2_seq1; B, comp87700_c1_seq4; C, comp63034_c0_seq1; D, comp78698_c0_seq1; E, comp65588_c0_seq1; F, comp69403_c0_seq1; G, comp47245_c0_seq1; H, comp60083_c0_seq1; I, comp87700_c0_seq2; J, comp37818_c0_seq1; K, comp29963_c0_seq1; L, comp20955_c0_seq1; and M, comp71879_c0_seq1, as detected by qRT-PCR.
