# Supplementary figures and images for: Comparative Transcriptome Analysis Reveals Critical Function of Sucrose Metabolism Related-Enzymes in Starch Accumulation in the Storage Root of Sweet Potato
Source: Front Plant Sci. 2017 Jun 22;8:914. doi: 10.3389/fpls.2017.00914 (PMC5480015; doi:10.3389/fpls.2017.00914)

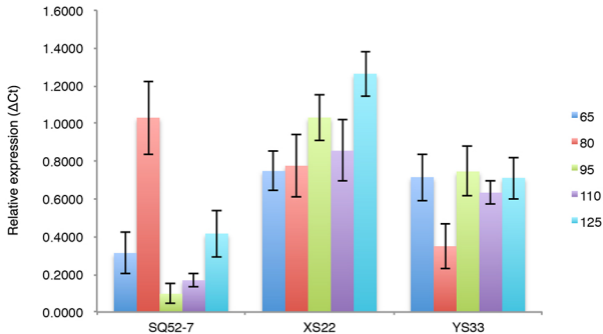

Figure S9 QRT-PCR detection of the expression patterns of genes encoding UGPase.

Supplement: Supplementary file 18 [file Image9.PDF]
