# Supplementary material for: Comparative Transcriptome Analysis Reveals Critical Function of Sucrose Metabolism Related-Enzymes in Starch Accumulation in the Storage Root of Sweet Potato
Source: Front Plant Sci. 2017 Jun 22;8:914. doi: 10.3389/fpls.2017.00914 (PMC5480015; doi:10.3389/fpls.2017.00914)
Supplement: Supplementary file 19 [file Image10.PDF]

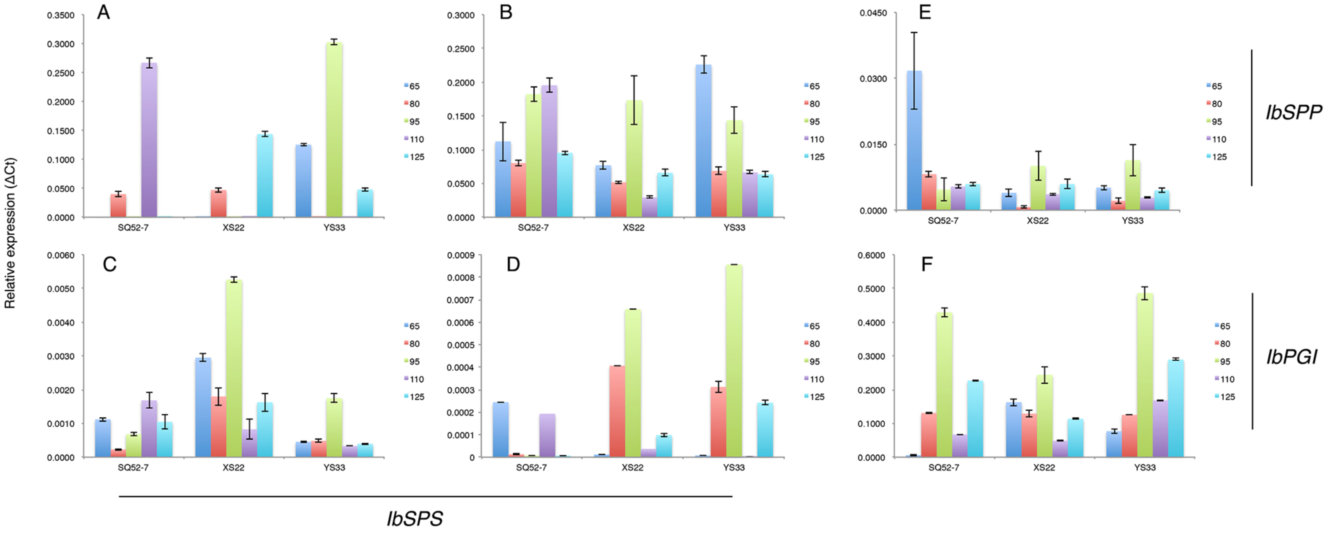

Figure S10 QRT-PCR detection of the expression patterns of genes encoding enzymes participating in sucrose metabolism during SR development in the three sweet potato genotypes examined.
A–D, the expression pattern of SPS encoding unigenes comp27340_c0_seq1, comp86708_c0_seq2, comp79328_c0_seq4, and comp72263_c0_seq1, respectively;
E and F, the expression pattern of SPP and PGI encoding unigenes comp81691_c1_seq1 and comp85765_c0_seq1, respectively.
