# Supplementary material for: Comparative Transcriptome Analysis Reveals Critical Function of Sucrose Metabolism Related-Enzymes in Starch Accumulation in the Storage Root of Sweet Potato
Source: Front Plant Sci. 2017 Jun 22;8:914. doi: 10.3389/fpls.2017.00914 (PMC5480015; doi:10.3389/fpls.2017.00914)
Supplement: Supplementary file 20 [file Image11.PDF]

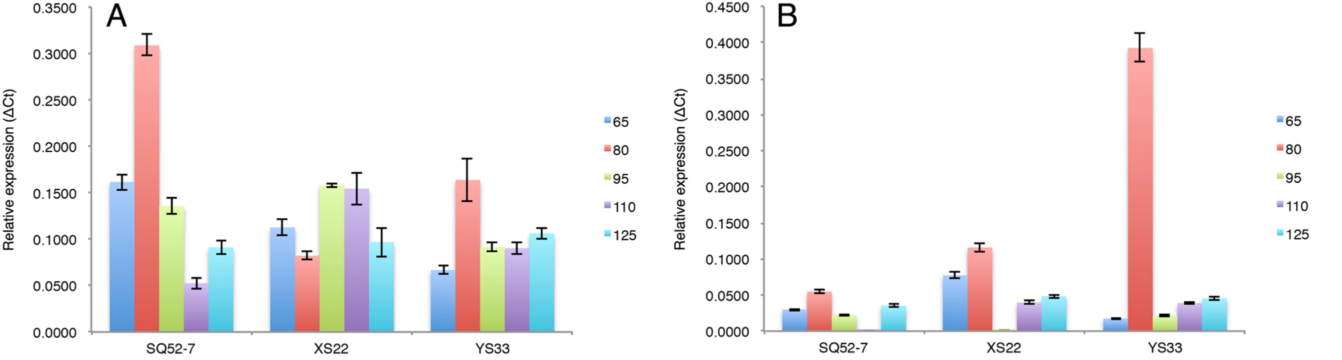

Figure S11 Expression patterns of unigenes encoding UGDH (A, comp87686_c0_seq4) and GAE (B, comp84725_c1_seq1), as detected by qRT-PCR.
