# Supplementary material for: Comparative Transcriptome Analysis Reveals Critical Function of Sucrose Metabolism Related-Enzymes in Starch Accumulation in the Storage Root of Sweet Potato
Source: Front Plant Sci. 2017 Jun 22;8:914. doi: 10.3389/fpls.2017.00914 (PMC5480015; doi:10.3389/fpls.2017.00914)
Supplement: Supplementary file 21 [file Image12.PDF]

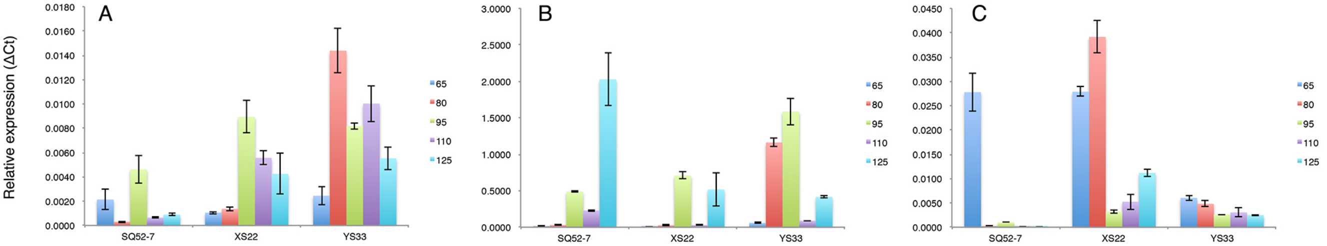

Figure S12 Expression patterns of invertase inhibitor genes, as determined by qRT-PCR.

A, B, and C, the expression pattern of unigenes comp70068_c0_seq1, comp59423_c0_seq1, and comp80373_c0_seq2, respectively.
