# Supplementary material for: Comparative Transcriptome Analysis Reveals Critical Function of Sucrose Metabolism Related-Enzymes in Starch Accumulation in the Storage Root of Sweet Potato
Source: Front Plant Sci. 2017 Jun 22;8:914. doi: 10.3389/fpls.2017.00914 (PMC5480015; doi:10.3389/fpls.2017.00914)
Supplement: Supplementary file 22 [file Image13.PDF]

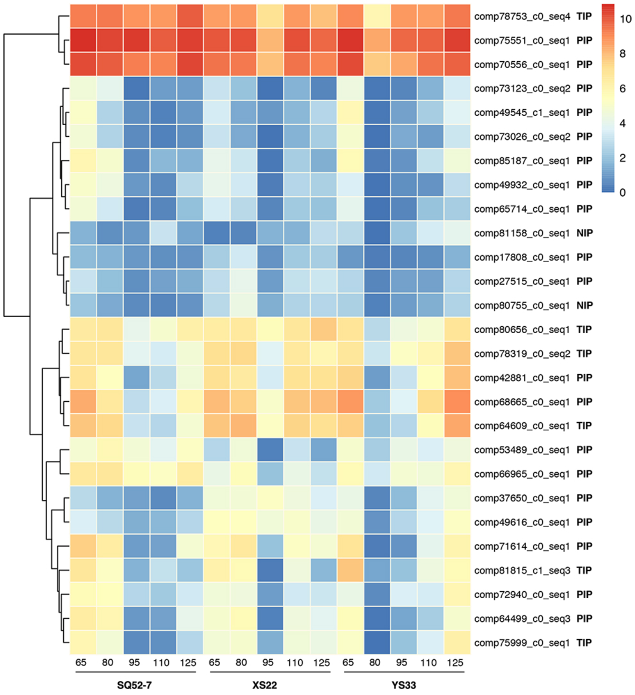

Figure S13 Heatmap showing expression patterns of aquaporin encoding unigenes.

Normalized log2 transformed RPKM gene expression values were used to plot the heatmap. PIP, TIP, and NIP, the plasma membrane intrinsic protein, tonoplast intrinsic protein, and Nodulin 26-like intrinsic membrane proteins, respectively.
