# Supplementary material for: Comparative Transcriptome Analysis Reveals Critical Function of Sucrose Metabolism Related-Enzymes in Starch Accumulation in the Storage Root of Sweet Potato
Source: Front Plant Sci. 2017 Jun 22;8:914. doi: 10.3389/fpls.2017.00914 (PMC5480015; doi:10.3389/fpls.2017.00914)
Supplement: Supplementary file 23 [file Image14.pdf]

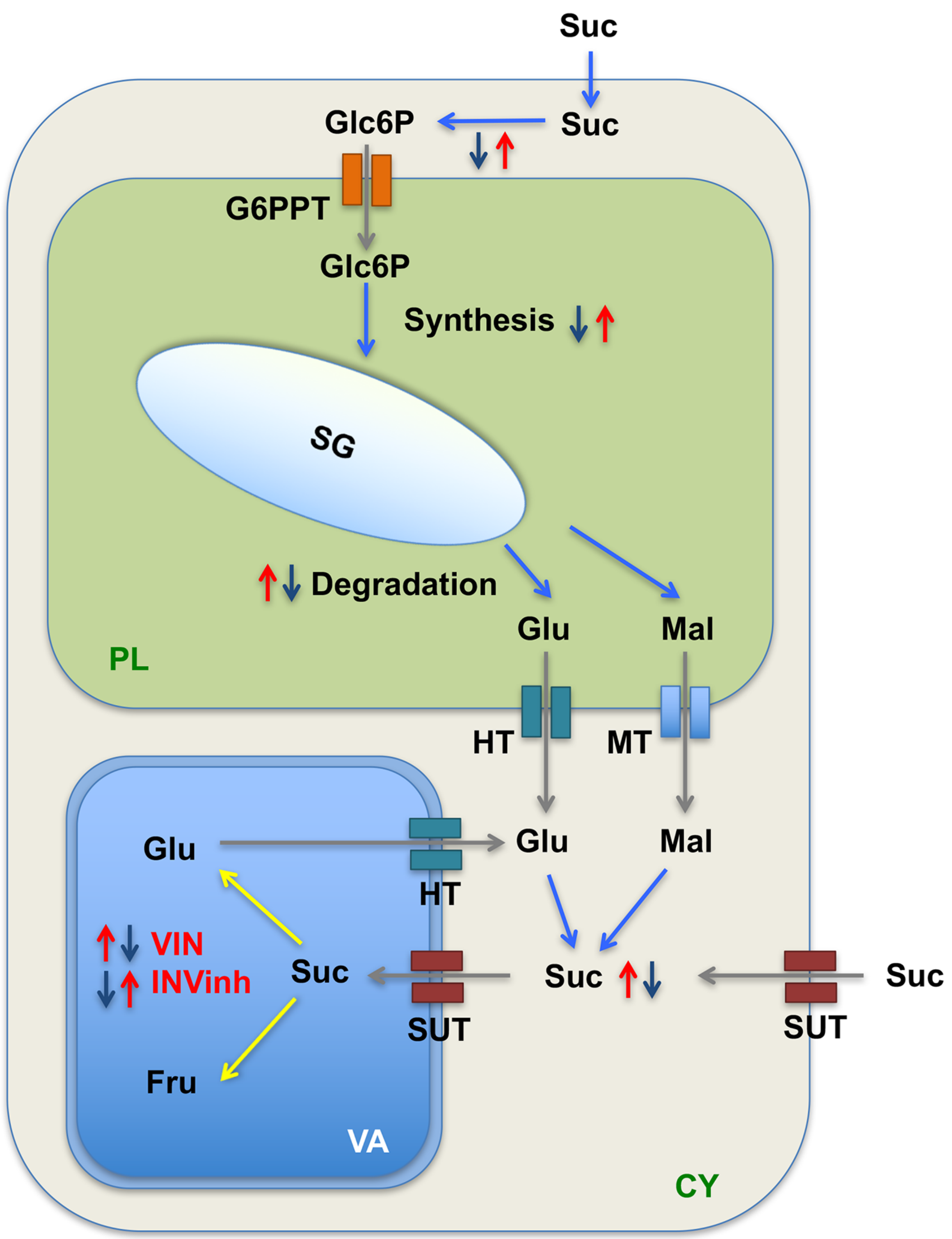

Figure S14 Scheme showing model in which vacuolar invertase regulates the starch content of sweet potato SRs.

Modified from Schreiber et al. (2014). CY, cytosol; Fru, Fructose; Glc6P, glucose-6-phosphate; Glu, Glucose; VIN, vacuolar invertase; INVinh, invertase inhibitor; Mal, maltose; PL, plastid; SG, starch granule; Suc, sucrose; and VA, vacuole. The rectangles at membranes indicate the following transporters: G6PPT, glucose-6-phosphate/ phosphate translocator; HT, hexose transporter; MT, maltose transporter; and SUT, sucrose transporter. Red and blue arrows indicate up- and down-regulation at the reaction step.
